# Data‐independent acquisition‐based SWATH‐MS for quantitative proteomics: a tutorial

**DOI:** 10.15252/msb.20178126

**Published:** 2018-08-13

**Authors:** Christina Ludwig, Ludovic Gillet, George Rosenberger, Sabine Amon, Ben C Collins, Ruedi Aebersold

**Affiliations:** ^1^ Bavarian Center for Biomolecular Mass Spectrometry (BayBioMS) Technical University of Munich (TUM) Freising Germany; ^2^ Department of Biology Institute of Molecular Systems Biology ETH Zurich Zurich Switzerland; ^3^ Department of Systems Biology Columbia University New York NY USA; ^4^ Faculty of Science University of Zurich Zurich Switzerland

**Keywords:** data‐independent acquisition, mass spectrometry, quantitative proteomics, SWATH‐MS, systems biology, Genome-Scale & Integrative Biology, Methods & Resources, Post-translational Modifications, Proteolysis & Proteomics

## Abstract

Many research questions in fields such as personalized medicine, drug screens or systems biology depend on obtaining consistent and quantitatively accurate proteomics data from many samples. SWATH‐MS is a specific variant of data‐independent acquisition (DIA) methods and is emerging as a technology that combines deep proteome coverage capabilities with quantitative consistency and accuracy. In a SWATH‐MS measurement, all ionized peptides of a given sample that fall within a specified mass range are fragmented in a systematic and unbiased fashion using rather large precursor isolation windows. To analyse SWATH‐MS data, a strategy based on peptide‐centric scoring has been established, which typically requires prior knowledge about the chromatographic and mass spectrometric behaviour of peptides of interest in the form of spectral libraries and peptide query parameters. This tutorial provides guidelines on how to set up and plan a SWATH‐MS experiment, how to perform the mass spectrometric measurement and how to analyse SWATH‐MS data using peptide‐centric scoring. Furthermore, concepts on how to improve SWATH‐MS data acquisition, potential trade‐offs of parameter settings and alternative data analysis strategies are discussed.

## Introduction

Over the last decades, liquid chromatography coupled to tandem mass spectrometry (LC‐MS/MS) has become the technology of choice for the high‐throughput characterization of proteins and proteomes (Aebersold & Mann, 2016). Recent developments in the field have moved beyond enumerating the proteins, peptides or post‐translational modifications detected in one or few samples towards delivering high quality and consistent quantification in large‐scale projects that comprise 100s of samples. Especially in areas such as personalized medicine, biomarker research, drug screens, genetic association studies or systems biology, large numbers of individuals, conditions and perturbations need to be investigated to draw meaningful biological conclusions. For this purpose, the large data matrices generated must be as reproducible, complete and accurate as possible. In order to address these needs, several different proteomic strategies have been developed over the last years.

**Figure 1:**
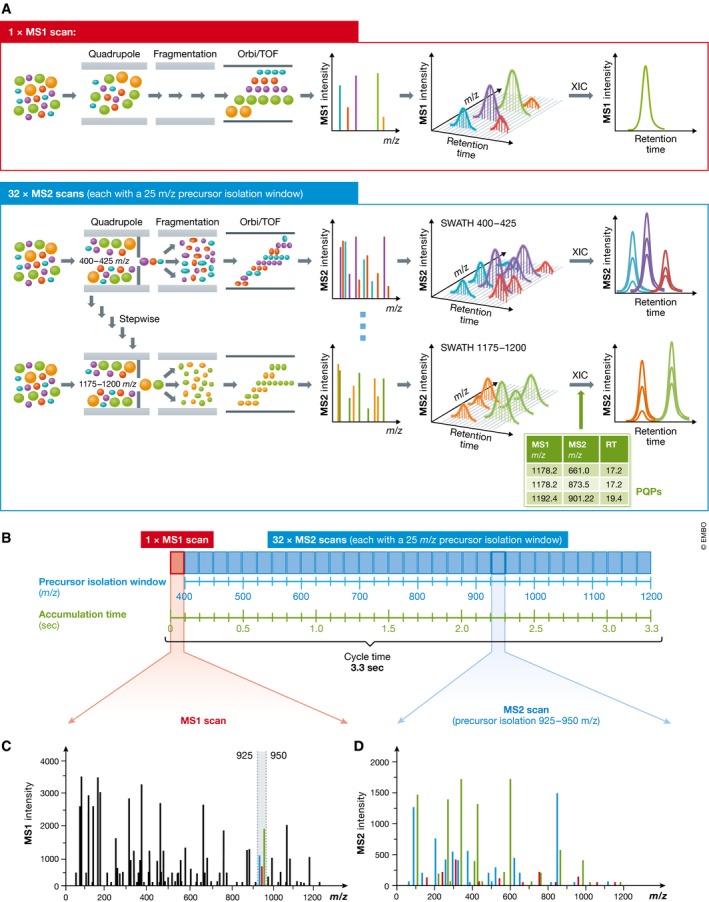
Principle of sequentially windowed data‐independent acquisition in SWATH‐MS (A) SWATH‐MS measurements are performed on fast scanning hybrid mass spectrometers, typically employing a quadrupole as first mass analyser and a TOF or Orbitrap as second mass analyser. In SWATH‐MS mode, typically a single precursor ion (MS1) spectrum is recorded, followed by a series of fragment ion (MS2) spectra with wide precursor isolation windows (for example 25 *m/z*). Through repeated cycling of consecutive precursor isolation windows over a defined mass range, a comprehensive data set is recorded, which includes continuous information on all detectable fragment and precursor ions. Hence, extracted ion chromatograms can be generated on MS2 as well as MS1 level. For the analysis of SWATH‐MS data, a peptide‐centric scoring strategy can be employed, which requires prior knowledge about the chromatographic and mass spectrometric behaviour of all queried peptides in form of peptide query parameters (PQPs). (B) The SWATH‐MS data acquisition scheme described by Gillet *et al* (2012) for a Q‐TOF mass spectrometer uses 32 MS2 scans with defined increments of 25 *m/z*, starting at 400 *m/z* and ending at 1,200 *m/z*. One full MS1 scan is recorded at the beginning. By applying an acquisition time of 100 ms per scan, a total cycle time of ~3.3 s is achieved. (C) The MS1 full scan detects all peptide precursors eluting at a given time point. For example, in the mass range from 925 to 950 *m/z,* three co‐eluting peptide species are detected (green, red and blue). (D) The corresponding MS2 scan with a precursor isolation window of 925–950 *m/z* represents a mixed MS2 spectrum with fragments of all three peptide species.

An emerging strategy, and the focus of this tutorial, is sequential window acquisition of all theoretical mass spectra (SWATH*‐*MS), which was described by Gillet *et al* (2012). For a detailed introduction into the history, the basic principles as well as the general advantages and limitations of SWATH‐MS (also summarized in Table 1), we refer to the Appendix. Briefly, for a SWATH‐MS measurement, typically non‐labelled protein samples are digested with trypsin and the resulting peptides are analysed by liquid chromatography coupled to a tandem mass spectrometer operating in the so‐called data‐independent acquisition (DIA) mode. In this mode, all ionized compounds of a given sample that fall within a specified mass range are fragmented in a systematic and unbiased fashion. Figure 1A–C shows the DIA scheme described as the initial implementation of SWATH‐MS (Gillet *et al*, 2012), using 32 consecutive, slightly overlapping precursor isolation windows, with a width of 25 *m/z* each. Depending on sample complexity, this acquisition scheme will lead to the co‐fragmentation of many co‐eluting peptides concurrently selected in the precursor ion window and ultimately to highly multiplexed and complex fragment ion spectra (Fig 1D). To deal with this complexity, Gillet *et al* proposed a novel data analysis strategy based on peptide‐centric scoring, which relies on querying chromatographic and mass spectrometric coordinates of the proteins and peptides of interest in form of so‐called peptide query parameters (PQPs). PQPs are typically derived from previously generated spectral libraries.

**Table 1:**
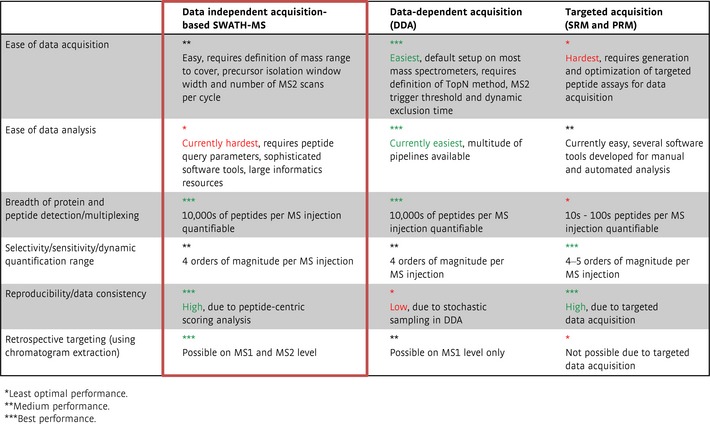
Advantages and limitations of SWATH‐MS in comparison with data‐dependent (DDA) and targeted (SRM, PRM) proteomics

In addition to the described SWATH‐MS method, a wealth of other DIA schemes and alternative data analysis strategies has been established. For an overview, see Appendix Fig S1, as well as recent reviews (Chapman *et al*, 2014; Bilbao *et al*, 2015). While the term “SWATH” became a registered trademark of SCIEX in the context of Q‐TOF instrumentation, the company Biognosys trademarked the name “Hyper Reaction Monitoring” (HRM) for an analogous mode of data acquisition on Orbitrap instrumentation (Box 1). Throughout this tutorial, we only use the term “SWATH‐MS”, independently from the underlying instrument type, and the generic term “DIA” when referring to the breadth of all data‐independent acquisition strategies.

**Box 1: Definitions of frequently used terms in the context of SWATH‐MS**

**Table:** 

Term	Definition
DIA	Data‐Independent Acquisition (DIA)—here, we use DIA as an umbrella term for mass spectrometric acquisition methods that continuously acquire fragment ion (MS2) spectra in an unbiased fashion, without requiring the detection of peptide precursor ions in an MS1 survey scan (as in DDA) nor prior knowledge about peptide precursor *m/z* values (as in SRM and PRM). Specific acquisition methods of the DIA family include for example SWATH‐MS, Shotgun‐CID, MS^E^, XDIA, MSX, AIF FT‐ARM and others
SWATH‐MS	Sequential Windowed Acquisition of All Theoretical Fragment Ion Mass Spectra (SWATH‐MS)—represents a specific variant of DIA, performed on hybrid full‐scan instruments (preferably Q‐TOF and Q‐Orbitrap). The term SWATH is a registered trademark of SCIEX. In SWATH‐MS data acquisition, successive pre‐defined ranges of precursor *m/z* values are isolated and subjected to co‐fragmentation (Gillet *et al*, 2012). Peptide‐centric scoring of SWATH‐MS data can be performed for example by using prior knowledge in form of a spectral library
HRM	Hyper Reaction Monitoring (HRM)—Synonym for SWATH‐MS. The term has been implemented in the context of data acquisition on Orbitrap mass analysers and is a registered trademark of Biognosys
Peptide‐centric scoring	A data query strategy which starts with a pre‐defined list of target peptides and tests whether those peptides are detectable in the data with a certain confidence. It can be applied to individual MS2 spectra or to extracted ion chromatograms (XICs). For a peptide‐centric scoring analysis, peptide query parameters need to be readily available. Typically, data acquired in SRM or PRM mode are analysed by peptide‐centric scoring, but also data acquired by DIA methods, such as SWATH‐MS, can be analysed in this way. An equivalent term also used in the literature is “targeted data extraction”
Spectrum‐centric scoring	A data analysis type which aims at finding the peptide sequence(s) from a user‐specified proteome that explain(s) a given MS2 spectrum best. It is typically applied in the context of discovery‐driven proteomics with data acquired by data‐dependent acquisition (DDA), but also data acquired by different DIA methods can be analysed in this way.
Peptide query parameters (PQPs)	Compendium of parameters required for peptide identification by peptide‐centric scoring. PQPs are stored in a table format and include (i) optimal (proteotypic) peptides to target for a given protein, (ii) chromatographic elution times of those peptides on the applied chromatography setup, (iii) most intense fragment ions (typically four to six) generated under the applied fragmentation conditions, (iv) charge state(s) of precursors and fragment ions and (v) relative ion intensity of all selected fragments. PQPs can be derived from previous discovery‐driven experiments, from which all peptide identifications are summarized in form of a spectral library
(Targeted) Peptide assay	Synonym for peptide query parameters (PQPs). Term mainly used in the context of SRM, where different tier levels (1–3) of analytical assay validation have been defined (Carr *et al*, 2014)
Spectral library	Compendium of MS2 spectra confidently assigned to a specific peptide sequence, typically acquired by discovery‐driven proteomics using data‐dependent acquisition. In case, several MS2 spectra refer to the same peptide sequence either the best scoring spectrum or an average consensus spectrum gets reported. Peptide retention time information can also be stored in a spectral library file and normalized retention times can be generated through retention time re‐alignment using reference peptides. Alternatively, spectral libraries can be generated from deconvoluted pseudo‐MS/MS spectra directly from DIA data (DIA‐Umpire; Tsou *et al*, 2015)
Transition	Pair of a precursor and one corresponding fragment ion *m/z* value. Mainly used in the context of SRM
Targeted proteomics	Umbrella term for mass spectrometric methods that aim at quantifying a list of pre‐defined proteins, peptides or PTM‐peptides of interest. In the two classical targeted proteomic approaches, SRM and PRM, the data acquisition itself is performed in a targeted fashion. However, also data acquired “untargeted”, using for example DIA measurements, can be analysed using a peptide‐centric data analysis strategy, which classifies SWATH‐MS as targeted proteomic approaches

### When is SWATH‐MS the method of choice for my proteomic study?

The major advantage of SWATH‐MS is that it supports quantitative analyses of peptides covering 1,000s of proteins with a high quantitative consistency and accuracy. It is ideally suited for projects that entail a large number of samples and that require accurate and reproducible quantification for the major fraction of the expressed proteome or peptidome in each sample. Typical projects that require exactly these properties include for example biomarker studies (Liu *et al*, 2014; Muntel *et al*, 2015; Kulkarni *et al*, 2016; Ortea *et al*, 2016), genetic association studies (Liu *et al*, 2015; Okada *et al*, 2016; Williams *et al*, 2016), clinical drug/perturbation studies (preprint: Litichevskiy *et al*, 2018; Tan *et al*, 2017; Keam *et al*, 2018) or exploratory basic research (Collins *et al*, 2013; Lambert *et al*, 2013; Parker *et al*, 2015a; Schubert *et al*, 2015b). SWATH‐MS is also particularly well suited for studies that need fast analyses using LC gradient lengths below 60 min (Vowinckel *et al*, 2018). Proteome coverages of ~50% of the MS‐detectable proteome have been achieved in complex mammalian samples in a single‐shot analysis (Bruderer *et al*, 2017; Kelstrup *et al*, 2018). A current drawback of SWATH‐MS compared to the classical targeted proteomic approaches (SRM or PRM) is that peptide quantification with SWATH‐MS is still three‐ to 10‐fold less sensitive (Gillet *et al*, 2012; Liu *et al*, 2013; Schmidlin *et al*, 2016). Hence, targeted data acquisition remains the better option for projects that involve quantification of particularly low‐abundant proteins and peptides with maximal accuracy. A further drawback of SWATH‐MS in comparison with DDA‐based methods is the required upfront effort on experimental or *in silico* spectral library and PQP generation and optimization (Table 1).

In the context of very large‐scale quantitative proteomic analyses, two alternative mass spectrometric strategies are currently used successfully in the field in addition to SWATH‐MS. The first is the classical label‐free DDA proteomics workflow, where quantification is based on precursor ion (MS1) intensities or spectral counts, and which can possibly be combined with peptide fractionation techniques to improve proteome coverage (Lawrence *et al*, 2015; Geyer *et al*, 2016; Frejno *et al*, 2017). An important improvement for the application of MS1 quantification to DDA data sets was the development of analysis tools that allow the transfer of peptide identifications between samples and thereby improve the completeness of the quantitative data matrix (Prakash *et al*, 2006; Mueller *et al*, 2007; Cox *et al*, 2014). However, even when using these tools, the number of missing values in DDA data sets still remains higher than for data acquired in SWATH‐MS mode, especially for peptides and proteins in the low concentration range (Bruderer *et al*, 2015, 2017; Kelstrup *et al*, 2018). While direct comparisons of an optimal label‐free MS1/DDA workflow versus a SWATH‐MS workflow are challenging, several papers have demonstrated that when the same sample is injected under the same conditions using the same mass spectrometer operated once in DDA and once in SWATH‐MS mode, SWATH‐MS outperforms DDA in terms of detectable peptides and associated proteins as well as measurement reproducibility (Bruderer *et al*, 2015; Kelstrup *et al*, 2018).

A second popular strategy for large‐scale quantitative proteomics relies on isobaric labelling, using, for example, tandem mass tags (TMT) (Thompson *et al*, 2003) or isobaric tags for relative and absolute quantitation (iTRAQ) (Unwin *et al*, 2005). Frequently, isobaric labelling is followed by an extensive peptide fractionation procedure. The resulting fractions are then analysed individually by DDA mass spectrometry (Chick *et al*, 2016; Roumeliotis *et al*, 2017). With state‐of‐the‐art TMT reagents 10 (McAlister *et al*, 2012) or 11, samples can be mixed and analysed simultaneously, leading to minimal sample preparation biases and highly consistent and complete data matrices within a set of multiplexed samples. While early implementations of this method suffered from quantitative ratio compression due to interferences in reporter ions from co‐eluting and co‐fragmenting peptides, this has been addressed to some extent by using optimized data acquisition and analysis methods (Ting *et al*, 2011; Savitski *et al*, 2013; McAlister *et al*, 2014; Ahrne *et al*, 2016; O'Brien *et al*, 2018; Sonnett *et al*, 2018). Labelling samples with isobaric tags can be an optimal workflow for comparative analysis of medium‐sized projects; however, if 100s of samples need to be analysed, the issues of data incompleteness and batch effects can become apparent again across sets of multiplexed samples. To date, studies that directly compare isobaric tagging with SWATH‐MS are missing.

The overall intent of this tutorial is to guide readers towards performing their own SWATH‐MS measurements. We give guidelines on how to set up and plan a SWATH‐MS experiment, how to perform the mass spectrometric measurement using data‐independent acquisition and how to analyse SWATH‐MS data using peptide‐centric scoring. Furthermore, concepts on how to improve SWATH‐MS data acquisition, potential trade‐offs of parameter settings and alternative data analysis strategies are discussed.

## Setting up and planning a SWATH‐MS experiment

If a SWATH‐MS study progresses towards the analysis of 100s and eventually 1,000s of samples, particular attention should be paid to the feasibility of producing comparable data of good quality, both longitudinally on a single instrument, as well as across multiple instruments of the same type, and conceivably across different instrument platforms. Encouraging progress has been made demonstrating the comparability of SWATH‐MS data generated between laboratories using standard samples (Collins *et al*, 2017).

Cumulative instrument contamination during a measurement series, caused for example by contaminants such as lipids, polymers or detergents, is a major concern and extra care must be taken to produce samples that are mostly devoid of such contaminants. Contaminants are an especially important issue in the context of SWATH‐MS, because we observed faster and more severe instrument performance loss (such as charging effects and sensitivity issues) in SWATH‐MS mode than in DDA or PRM mode on the same instrument. One possible explanation for this observation is that the instrument operated in SWATH‐MS mode has a substantially higher ion flux in the fragment ion scans (which compose > 90% of the data acquisition time), which in turn means that also more sample contaminants and impurities might enter and contaminate the instrument, leading to a faster performance loss. Therefore, monitoring the performance of the mass spectrometer and maintaining it at an acceptable level are an important prerequisite. Recent efforts have been undertaken to develop software tools that enable systematic tracking of instrument performance and that can be applied also to SWATH‐MS data (Rudnick *et al*, 2010; Wang *et al*, 2014; Bereman *et al*, 2016; Chiva *et al*, 2018).

When planning a label‐free large‐scale proteomic experiment, statistical considerations for the experimental design, such as group size, biological and technical variability or achievable sensitivity and selectivity, should be taken into account (Krzywinski & Altman, 2014b). We suggest that particular attention should be paid to proper randomization and blocking (Krzywinski & Altman, 2014a) of a sufficient number of biological and technical sample replicates (Blainey *et al*, 2014) to ensure optimal statistical power (Krzywinski & Altman, 2013) during the downstream data analysis process.

### Prior knowledge required for peptide‐centric scoring and spectral libraries

The underlying concept behind SWATH‐MS is that empirically derived prior knowledge of the mass spectrometric and chromatographic behaviour of peptides of interest can be used to selectively extract peptide‐specific information from highly convoluted SWATH data in a targeted fashion (Gillet *et al*, 2012). This required prior knowledge is referred to as “peptide query parameters” (PQPs). It is worth noting that “prior” in this context indicates that PQPs should be available as a prerequisite before data analysis is undertaken, while the actual acquisition of the SWATH‐MS data itself does not depend on the availability of PQPs.

**Figure 2:**
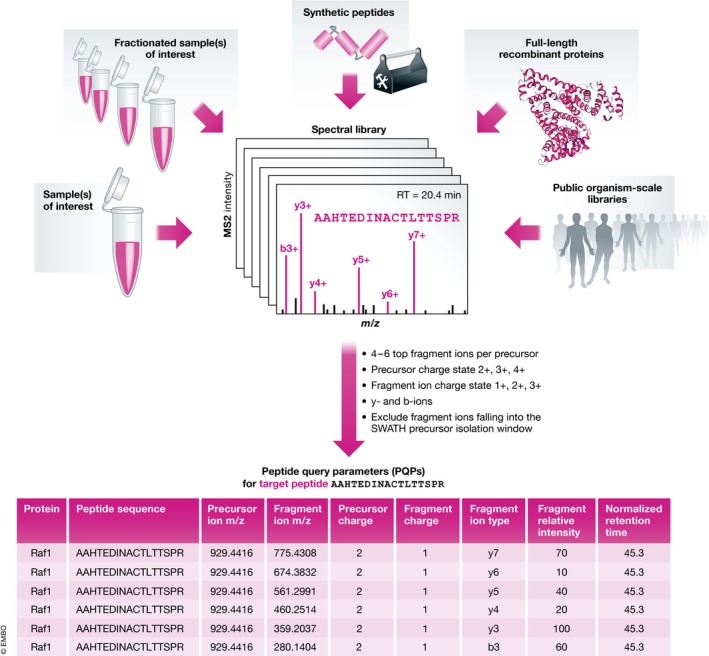
What are peptide query parameters (PQPs) and where do these parameters come from? PQPs contain information about the chromatographic and mass spectrometric behaviour of a given peptide, as exemplified here for the peptide AAHTEDINACTLTTSPR. Various different input sample types can be used for the purpose of PQP generation. Typically, those samples are analysed in initial DDA measurements, and the results are summarized in the form of one or several spectral library files. From the spectral library file(s), the relevant PQPs are extracted by filtering the identified peptide coordinates using the indicated criteria. PQPs contain information about: the underlying protein, peptide sequence, precursor *m/z*, fragment *m/z*, precursor and fragment charge, fragment ion type, expected relative fragment ion intensities and normalized retention time (retention time relative to a set of reference peptides, iRT).

What kind of information do PQPs contain? In detail, the information includes (i) the peptide sequence(s) to monitor for a given protein, (ii) the dominant precursor ion *m/z* value(s) of the peptide(s) and thus the charge state distribution, (iii) the four to six most intense fragment ion *m/z* values for the peptide(s) under the applied fragmentation conditions, (iv) information about the expected fragmentation pattern under the applied conditions, i.e. the relative fragment ion intensities, and v) the expected retention time of the peptide(s) and thus the associated fragment ion signals, ideally normalized to a reference. PQPs can commonly be obtained from a spectral library [or potentially chromatogram library (Sharma *et al*, 2014)] and are stored in a table format as shown in Fig 2. Computational pipelines integrating all steps of spectral library generation and PQP extraction have been developed that simplify and standardize this process and are available for example within Skyline (Egertson *et al*, 2015), PeakView (SCIEX), Spectronaut Pulsar (further referred to as Spectronaut) (Bruderer *et al*, 2015) (Biognosys) and the Trans‐Proteomic Pipeline (TPP) (Deutsch *et al*, 2010). Further tools to prepare and convert [specL (Panse *et al*, 2015), Fraggle/Tramler/Franklin (Teleman *et al*, 2017)] or extend [SwathXtend (Wu *et al*, 2016)] sets of peptide query parameters are also available. Particularly large spectral libraries can be optimized and constrained by using MSPLIT‐DIA (Wang *et al*, 2015).

For further details on how to create spectral libraries and PQPs from DDA data, we refer to a recent protocol paper (Schubert *et al*, 2015a). In the following paragraphs, we will discuss the types and sources of information that can be used to generate spectral libraries from which PQPs can be extracted.

An alternative to acquiring DDA runs for spectral library generation represents spectrum‐centric scoring of the DIA data. While such algorithms have been proposed early in the development of DIA acquisition schemes, recently developed algorithms such as DIA‐Umpire (Tsou *et al*, 2015), Group‐DIA (Li *et al*, 2015) and Spectronaut Pulsar (Biognosys) make specifically use of the improved data quality of the latest generation instruments. Generally, these algorithms generate a cumulative spectral library of a related set of samples and achieve similar coverage as DDA runs of unfractionated samples.

### Representative sample types for spectral library generation

Here, one has to choose between endogenous sources of peptides from the samples of interest, synthetic analogues of those peptides or recombinant full‐length proteins (Fig 2). The majority of SWATH‐MS studies to date have used a side‐by‐side characterization of the samples of interest by DDA analysis for generation of spectral libraries. These libraries frequently include a sample fractionation step prior to DDA analysis, which can be beneficial from a sensitivity perspective for post‐acquisition peptide queries (Rosenberger *et al*, 2014; Zi *et al*, 2014; Selevsek *et al*, 2015). This is because the sensitivity and coverage of single‐shot DDA analysis are frequently lower than that of SWATH‐MS data (Bruderer *et al*, 2015; Kelstrup *et al*, 2018). Therefore, the SWATH‐MS data would not be fully covered by a “single shot” DDA spectral library generation strategy. The strategy focusing on repeated DDA analysis of non‐fractionated samples is sometimes favoured because it is straightforward to implement; however, primarily for library completeness and quality reasons, other sources of peptides can be considered.

Chemically synthesized peptides have long been proposed as a source of prior knowledge, in particular, with respect to the development of SRM assays (Kuster *et al*, 2005; Picotti *et al*, 2010), and large‐scale efforts to synthesize and measure peptides for several organisms on a proteome‐wide scale have been reported (Picotti *et al*, 2013; Schubert *et al*, 2013; Kusebauch *et al*, 2016; Zolg *et al*, 2017). This approach has several advantages: (i) all proteins can be represented in the library, irrespective of whether they have been previously observed, (ii) high‐quality MS2 spectra and derived PQPs can be generated, because the synthetic peptides can be analysed at very high concentrations and represent ground truth, and (iii) the error rate in the spectral library generated should be close to zero. In some cases, the selection of which peptides to synthesize for a given protein has been driven by prior empirical observation. However, since there has been so far no species with absolute complete proteome coverage by DDA methods, sets of proteotypic peptides for each protein have also been computationally predicted (Mallick *et al*, 2007). Since computational prediction methods have turned out to be less reliable than expected (Searle *et al*, 2015), empirical peptide selection is still preferred over computational prediction. More recent efforts to create synthetic proteomes for general purposes in proteomics have extended to a very large scale (> 330,000 human peptides) by synthesizing much larger numbers of peptides per protein and by also including PTMs and common sequence variants (http://www.proteometools.org; Zolg *et al*, 2017). A useful extension of this approach is to create full‐length proteins by recombinant methods or *in vitro* transcription/translation systems. In this way, the most suitable peptides per protein for analysis can be determined empirically (Stergachis *et al*, 2011; Matsumoto *et al*, 2017). While those large‐scale synthetic peptide and protein MS resources have not yet been fully exploited for SWATH‐MS analysis, it seems likely that they will be useful resources going forward. Hybrid libraries consisting of endogenous samples and synthetic peptides to increase coverage may also be an attractive option and have proved useful in the case of an organism‐scale library for *Mycobacterium tuberculosis* (Schubert *et al*, 2015b).

### Comprehensive versus sample‐specific spectral libraries

A natural extension of the ideas discussed above is to attempt to characterize peptides by DDA from all proteins in a given species to generate a comprehensive “off the shelf”, species‐specific library for general use, obviating the need to generate experiment‐ or sample‐specific libraries. These libraries could include data from endogenous samples and/or synthetic proteomes. Presently, such organism‐scale spectral libraries are publicly available for *S. pyogenes* (Karlsson *et al*, 2012), *S. cerevisiae* (Picotti *et al*, 2013; Selevsek *et al*, 2015), *M. tuberculosis* (Schubert *et al*, 2013, 2015b), *H. sapiens* (Rosenberger *et al*, 2014; Kusebauch *et al*, 2016; Zolg *et al*, 2017), phyllosphere‐colonizing bacteria (*M. exotorquens*,* P. syringae* and *S. melonis*) (Muller *et al*, 2016) and *E. coli* (Ludwig *et al*, in preparation). Further, the number of public repositories for various types of MS data that could conceivably also be used as prior knowledge in the analysis of SWATH‐MS data continues to increase (Craig *et al*, 2004; Martens *et al*, 2005; Deutsch *et al*, 2008; Picotti *et al*, 2008; Sharma *et al*, 2014; Whiteaker *et al*, 2014; Wilhelm *et al*, 2014; Zolg *et al*, 2017). These include SWATHAtlas (http://www.swathatlas.org), which focuses on SWATH‐MS and provides spectral libraries in formats directly compatible with most software tools for peptide‐centric analysis of SWATH‐MS data.

An important consideration for the use of such public libraries will be portability of information between instrument types and between laboratories. For example, efforts have been made to evaluate the effect of using DDA spectra generated on different instrument types as prior knowledge (Toprak *et al*, 2014). Although the best comparability is achieved when fragment ion spectra are generated on the same instrument platform, the data also indicated that fragment ion spectra acquired from instruments using “beam type” collision‐induced dissociation (e.g. QqQ, QqTOF and Orbitrap operated in HCD mode) produce spectra that are sufficiently comparable for their effective use as prior knowledge in peptide‐centric analysis (de Graaf *et al*, 2011; Zolg *et al*, 2017). The portability of chromatographic information between laboratories has been advanced through the use of normalized retention times (Norbeck *et al*, 2005) such as indexed retention time (iRT) (Escher *et al*, 2012). The accuracy of such methods is generally tolerant to changes in gradient length and column dimensions; however, larger errors will result from changes in other factors that may significantly affect the peptide elution order such as mobile/stationary phases or column temperature. An inter‐laboratory evaluation study recently showed that an organism‐scale spectral library could effectively be used to analyse SWATH‐MS data generated from 11 different laboratories worldwide (Collins *et al*, 2017).

Another important issue to consider when working with very large‐scale spectral library resources and querying very high numbers of peptides in many samples measured by SWATH‐MS is appropriate error rate control. In such analyses, it is common that a significant fraction of queried peptides from the library are actually not present in the samples of interest at a detectable level and, as such, represent channels in which false positives may appear and accumulate if many runs are analysed. If SWATH extraction results are summarized at the inferred protein level, the error rate can be further inflated by such false positives (Reiter *et al*, 2009; Rosenberger *et al*, 2017a). Therefore, it is important to control false discovery rates at the protein level, rather or in addition to the peptide level and to compute false discovery rates globally for a complete experiment rather than at the per‐file level. Rosenberger *et al* (2017a) recently established statistical concepts derived from discovery proteomics that can be applied to appropriately control the error rate of detected peptides and inferred proteins in SWATH‐MS.

In conclusion, using organism‐scale “off the shelf” libraries might be convenient from the perspective of reducing the effort of creating project specific libraries, but their use may eventually result in some loss of statistical power due to the additional multiple testing burden and necessary correction that comes from querying such a large number of peptides. Alternatively, researchers need not necessarily query all of the peptides in a spectral library, but can also focus on just a subset of proteins that are of interest for their specific biological question.

### Which and how many peptides should be queried for?—From targeted to discovery uses of SWATH‐MS

In the initial implementation of SWATH‐MS, the primary goal was to use peptide‐centric scoring based on prior knowledge combined with sequentially windowed data‐independent acquisition (Gillet *et al*, 2012) to develop an approach that generates data resembling those from classical targeted proteomics approaches such as SRM but at a largely extended scale of analytes. Hence, a discrete set of peptides from proteins related to a particular biological question was targeted for quantification. However, it was soon realized that SWATH‐MS data sets offer the opportunity to characterize complex samples in a more comprehensive manner by extracting and scoring ion chromatograms for a maximum number of peptides, by using sample‐specific or combined libraries. Over time, the number of peptides that were queried in comprehensive SWATH‐MS studies has grown significantly to the point that, with the appropriate error‐rate control (Rosenberger *et al*, 2017a), it is now possible and reasonable to query for tens to hundreds of thousands of peptides derived from organism‐scale spectral libraries in a fashion that resembles discovery‐based proteomics much more than targeted proteomics in its scope (Bruderer *et al*, 2017).

The choice of whether to choose a targeted approach and extract peptides for only a smaller set of proteins of interest, or whether to extract data for all peptides contained in a large‐scale spectral library, will depend on the specificity of the hypothesis that underlies the targeted approach, i.e. how useful the set of proteins being measured is for answering a given biological question. If a biological hypothesis can be addressed with a selected set of target proteins, then increasing the number of queries by including peptides from a large‐scale library will increase the multiple testing burden. If the hypothesis cannot be addressed by few selected proteins and requires a data‐driven investigation, such as exemplified by classification problems, then extracting a maximum number of peptides from a large‐scale library will likely provide the most informative result, despite the increased number of statistical tests that need to be performed.

The possibility that specific peptide queries might result in false‐positive signals arising from contaminating peptide species that are similar to the target peptide in their mass spectrometric and chromatographic parameter space has been a concern of peptide‐centric scoring. This might be the case for peptides with a sequence that is similar to that of the target peptide, or for peptides for which the precursor mass difference between target and mismatch can be explained by a modification of the common backbone sequence. A recent study comparing standard database searching of DDA data with open modification searches of the same data found that this scenario occurs rather infrequently (Kong *et al*, 2017). The open modification search assigned a given fragment ion spectrum to a modified peptide sequence rather than to the unmodified peptide sequence originally assigned by the standard database search only for ca. 1% of the identified peptides. This suggests that false‐positive identifications in SWATH‐MS data due to unanticipated modified peptides might also occur rather infrequently, but nevertheless, close attention in ongoing algorithmic development should be paid to the likelihood of matching‐related peptide species (Rost *et al*, 2012). Data analysis methods have now been developed that address the problem of related “peptidoforms” in SWATH datasets (Rosenberger *et al*, 2017b).

Overall, the high degree of fragment ion data completeness, both in the mass and chromatographic dimension, constitutes a main strength of data acquired by SWATH‐MS and related DIA approaches, as it offers the opportunity of applying diverse and flexible data analysis strategies. It also equally supports different experimental strategies, including targeted quantification of selected analytes and discovery‐driven studies of all detectable analytes, provided that the types of possible scoring errors are recognized and appropriately controlled.

## Performing a SWATH‐MS measurement

### Setting up liquid chromatography for SWATH‐MS

The LC setup used for SWATH‐MS measurements does not differ from that used for targeted or DDA setups. Typically, peptide mixtures of 0.5–2 μg total peptide mass are separated by injection onto a nano‐HPLC system equipped with a 15–50‐cm‐long column packed with reversed phase particles (e.g. 3 or 1.8 μm C18 material), operated at 300 nl/min flow rate and eluted with a solvent gradient of increasing organic composition. Note that ~5 μl/min microflow HPLC systems are also compatible with SWATH‐MS, though at the cost of a three‐ to fivefold loss of sensitivity that can be compensated by injecting three to five times more peptide amount on column (Bruderer *et al*, 2017; Vowinckel *et al*, 2018).

**Figure 3:**
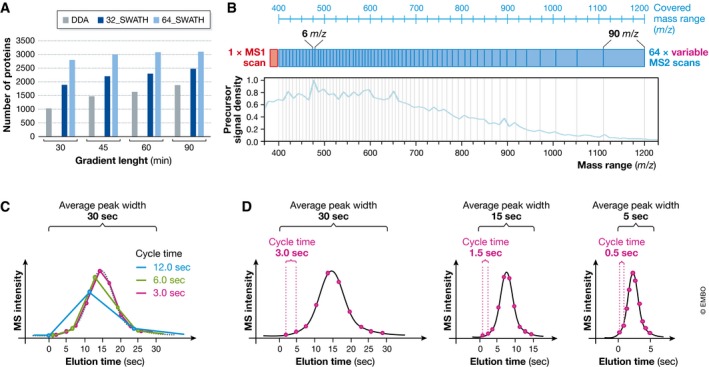
Setting up the optimal SWATH‐MS data acquisition scheme (A) Effect of liquid chromatography gradient length on the number of identified proteins for technical triplicate injections of a trypsin‐digested HEK cell lysate acquired either in DDA (grey bars), SWATH 32 fixed windows (dark blue bars) or SWATH 64 variable windows (light blue bars) on a Q‐TOF instrument. (B) To improve precursor selectivity in SWATH‐MS, an acquisition scheme using variable precursor isolation window widths can be used to partition the precursor density equally across all isolation windows. (C) Ten recorded data points are considered necessary for accurate reconstruction of a chromatographic peak. For a peak width of 30 s a cycle time of 3 s leads to 10 recorded data points (red), which allows appropriate reconstruction of the actual peak shape (grey dashed line). Longer cycle times, for example 6 s (green) or 12 s (blue), lead to under sampling and the correct peak shape can no longer be optimally reconstructed. (D) If the average peak width is reduced from 30 (left panel) to 15 (middle panel) or even 5 s (right panel), the cycle time needs to be decreased accordingly from 3 to 1.5 and 0.5 s, in order to maintain 10 data points over the elution profile.

Peptide‐centric analysis of SWATH‐MS data depends on a sufficient number of data points (typically in the range of 10) collected over the elution profile of a peptide peak to allow the accurate reconstruction of the respective chromatographic peak (Fig 3C). The 2–4 s cycle time required by the current windowed SWATH‐MS methods to cycle through the precursor isolation range, together with the required number of measurements over the peak indicates that the average chromatographic peak width in SWATH‐MS measurements should not fall below 20–40 s. This is in contrast to single‐window DIA methods, which only cycle between a single MS1 and a single MS2 scan and which can accommodate the 2–3 s‐wide chromatographic peaks produced by higher performance LC setups [for example AIF (Geiger *et al*, 2010) or MS^E^ (Silva *et al*, 2006)]. As MS instruments improve in scanning speed, narrower chromatographic peaks may increasingly become compatible with windowed SWATH‐MS methods.

Interestingly, SWATH‐MS copes well with shortening of the HPLC gradient length. While the number of acquired MS2 spectra and the number of peptides identified decrease proportionally with the gradient length in DDA mode, the deterministic nature of the MS2 sampling in SWATH‐MS mode attenuates the attrition in number of peptides identified for shorter separation gradients (Fig 3A). The loss in the number of peptide identifications at shorter gradients in SWATH‐MS mode seems, therefore, to be related to the increase in precursor co‐elution and ensuing potential ion suppression and co‐fragmentation effects rather than to undersampling. As MS instrumentation continues to improve, it is likely that SWATH‐MS will reach higher selectivity and identification rates under short gradient regimens compatible with relatively high sample throughput.

In summary, we advise a 2‐h nano‐HPLC gradient for the acquisition of high‐quality spectral libraries (Schubert *et al*, 2015a), while the use of shorter nano‐ (or micro‐) HPLC gradients to acquire SWATH‐MS data, for example in the range of 30–60 min, still provides results with good selectivity and proteome coverage at a significantly higher sample throughput.

### Setting up the SWATH‐MS data acquisition method

#### Finding an optimally balanced method

The optimal setup of precursor ion (MS1) and fragment ion (MS2) scans in a SWATH‐MS acquisition scheme depends on a range of considerations, including the expected mass range for analytes of interest, underlying sample complexity, available mass spectrometer type with its specific resolution and scanning speed, the LC setup and its expected average chromatographic peak width, as well as the desired measurement selectivity and sensitivity. In practice, the following instrument parameters need to be considered: (i) the precursor *m/z* range to cover, (ii) the widths and number of precursor isolation windows, (iii) the fragment and precursor ion accumulation/scanning time and resolution, (iv) the chromatographic cycle time and (v) the number of injections per sample. In the following section, each of these parameters is discussed in detail. The experience of the authors is primarily focussed on Q‐TOF instrument configurations, and our recommendations in that respect are based on empirical knowledge, optimization and simulation (Rost *et al*, 2012). However, as the quadrupole‐Orbitrap is increasingly popular for SWATH‐MS analysis, we also refer to successfully published methods on that configuration. A detailed overview of acquisition parameters for Q‐TOF as well as Q‐Orbitrap instruments is provided in Table EV1. Those parameters can serve as a starting point for the generation of SWATH‐MS data of good quality; however, further optimization might allow an increase in the level of quantifiable proteins and peptides. Of final note, optimal SWATH‐MS parameters for Q‐TOF as well as Q‐Orbitrap instruments are still subject to adaptations and might change significantly in the future.

##### Precursor *m/z* range to cover

The precursor *m/z* range covered in a SWATH‐MS measurement is defined by the adjacent set of precursor isolation windows that the instrument cycles through across the chromatographic separation. Ideally, this mass range should cover as completely as possible the *m/z* space of the proteins or peptides of interest. Analytes outside the specified range are not monitored and can therefore neither be detected nor quantified. If the aim is to analyse a significant portion of the proteome and if trypsin is used as proteolytic enzyme during the sample preparation, the most peptide‐rich region typically spans 400–1,200 *m/z* (Gillet *et al*, 2012). The range can be further reduced to 500–900 *m/z* (Egertson *et al*, 2015) depending on instrument and analyte constraints with a minimal attrition of quantified proteins. If other proteases than trypsin are used, or if SWATH‐MS is applied to analytes other than peptides (e.g. metabolites), the precursor *m/z* range needs to be adapted accordingly. Coordinates for *m/z* ranges successfully used in selected publications on Q‐TOF and Q‐Orbitrap instrument are given in Table EV1.

##### Precursor isolation window placement

The precursor isolation window width defines the range of peptide precursor masses that are co‐isolated and co‐fragmented in a given MS2 scan. Hence, the isolation window width directly influences the selectivity and the dynamic range of the measurement and in turn the sensitivity of peptide detection. It is probably the parameter that varies the most between various DIA methods, ranging from one isolation window of 800 *m/z* to hundred windows of 2 *m/z* (Appendix Fig S1).

Whereas the use of wider isolation windows allows the mass spectrometer to cycle faster through the predetermined precursor *m/z* range, it results in a higher number of peptide precursors being co‐fragmented, more strongly convoluted MS2 spectra and lower sensitivity due to a limited intra‐scan dynamic range. Conversely, using narrower isolation windows reduces the number of co‐fragmented precursors and signal interference, but limits other parameters such as the covered precursor *m/z* range per injection or the number of data points recorded over the chromatographic peak profile.

Note that the precursor isolation window width does not need to be identical for all MS2 scans. Rather, variable window widths (Zhang *et al*, 2015) can be used to optimize the number or intensity distribution of precursor ion signals between isolation windows (Fig 3B). Such optimal variable window patterns can be applied on a per‐sample basis or identically across multiple samples. Automated tools are available to facilitate such placement based on empirical data (“SWATH Variable Window Calculator” provided by SCIEX). In practice, we observed that the fine adjustment of the isolation window widths does not drastically influence the peptide identification or quantification results. For practical reasons, we therefore generated a “universal 64 variable window” acquisition scheme for TOF instruments (Table EV1) that seem compatible with most complex tryptic digests from various organisms ranging in complexity from bacteria to human. This precursor isolation scheme was devised to split ~50,000 experimental human tryptic precursor signals from a human cell line in 64 equal bins that vary from 5.9 *m/z* width for the range [472.4–478.3], where the density of precursor ion signals is the highest, up to 90.9 *m/z* width for the range [1,109.6–1,200.5]. The gain achieved by moving from 32 fixed to 64 variable windows has been demonstrated both qualitatively and quantitatively (Navarro *et al*, 2016). Bruderer *et al* optimized a SWATH‐MS acquisition method for the Q‐Exactive with 19 variable windows (Bruderer *et al*, 2015) and the Q‐Exactive HF with 24 variable windows (Bruderer *et al*, 2017), whereas Kelstrup *et al* (Kelstrup *et al*, 2018) published a method with 70 windows for the latest and fastest Q‐Exactive HF‐X instrument (Table EV1).

Recently, alternative DIA acquisition methods have reported the use of deterministic but differential isolation windows during LC separation and a peptide's elution profile (Egertson *et al*, 2013; Moseley *et al*, 2018). However, their analysis requires specific deconvolution tools to decipher, post‐acquisition, the contribution of the differential interferences arising during the peptide co‐isolation process across the various isolation schemes.

##### Fragment and precursor ion accumulation time/resolving power

For Q‐TOF instruments, the MS acquisition or accumulation time defines how long the mass spectrometer accumulates ion signals for a given MS scan. With longer accumulation times, the signal‐to‐noise ratio for the acquired spectrum increases. Conversely, longer accumulation times increase the time the instrument requires to cycle through the series of MS scans. Therefore, the accumulation time should be chosen in conjunction with the number of precursor isolation windows (Fig 3C).

For Orbitrap mass analysers, the MS acquisition is subdivided into two parallelized time‐constrained processes. First, the “injection time” is the time required to collect in the ion trap the desired number of charged species designated by the automatic gain control (AGC) parameter. Second, the “scan time” is the time required by the Orbitrap to record the mass spectrum depending on the set resolution. On new generation instruments (Q‐Exactive and Fusion/Lumos), these two processes are parallelized, and thus, the more time‐consuming step determines the cycle time. Typically, the maximal injection time is set according to the required scan time. Recent publications have reported methods with 19 (Bruderer *et al*, 2015) (Q‐Exactive) and 24 variable precursor isolation windows (Bruderer *et al*, 2017) (Q‐Exactive HF) at a resolution of 30,000 (at Full Width Half Maximum) or 70 windows (Kelstrup *et al*, 2018) (Q‐Exactive HF‐X) with fixed width of 9 *m/z* at a resolution of 15,000 (Table EV1). With the rapidly evolving specifications of these instruments, further optimization of the acquisition settings can be expected.

In summary, for Q‐TOF instruments coupled to a nano‐LC setup that results in an average chromatographic peak width in the range of ca. 30 s, we recommend to use an MS2 accumulation time in the range of 50 ms for each of 64 variable MS2 acquisition windows. This adds up to a total cycle time of ~3.3 s. On the Q‐Exactive HF instrument, the use of 24 variable windows at 30,000 resolution has been described recently, leading to a similar cycle time (Bruderer *et al*, 2017). However, due to the added complexity of balancing automatic gain control, scan time and duty cycle effects on Orbitrap instrumentation, further optimization for given instrument configurations and sample types should be considered. We also recommend the inclusion of a 250 ms MS1 scan on Q‐TOF instruments before each cycle of MS2 scans. Similarly, the inclusion of one or several MS1 scans is recommended for Orbitrap instruments. Information from the MS1 scan is increasingly being used by recent DIA analysis software, either to confirm the precursor mass of the peptide of interest upon peptide‐centric targeted chromatogram extraction or to aid in the deconvolution of co‐eluting fragment ion traces with spectrum‐centric analysis tools.

##### Chromatographic cycle time

In SWATH‐MS, the “cycle time”, also referred to as “duty cycle” or “sampling rate”, corresponds to the sum of the accumulation times set for the MS1 and MS2 scan series (plus any instrument overhead time which is usually negligible). Hence, the cycle time defines how often along the chromatographic elution profile of a peptide the same ion gets recorded. The cycle time is critical for any chromatography‐based quantification approach. Generally, around 10 acquired data points are considered necessary to reconstruct chromatographic peak shapes and to perform accurate quantification (Fig 3C) (Matthews & Hayes, 1976; Lange *et al*, 2008). However, the available time to collect 10 data points throughout an average chromatographic peak width can vary substantially, depending on the specific chromatographic setup. In an experiment with an average peak width (at base) in the range of 30 s (typical peak width in a 3 μm C18 particles nano‐HPLC setup with gradient length in the range of 1–2 h), a cycle time of 3 s is appropriate. However, if a higher resolution chromatographic system is employed, the average peptide elution peak width might be significantly shorter, for example 5 s. In such cases, the cycle time must be decreased, e.g. to 0.5 s, to ensure the constant recording of 10 data points over the elution profile (Fig 3D).

##### Number of injections per sample

For large‐scale proteomic studies that include 100s of samples, a single injection per sample is strongly preferable to maintain sample throughput and quantification consistency. However, for smaller scale projects, multiple injections per sample (*N*), each covering a different *m/z* range, might be an attractive alternative to improve selectivity and sensitivity of the SWATH‐MS measurement (Panchaud *et al*, 2009; Ting *et al*, 2017). This allows the reduction in precursor isolation window width by a factor of *N*, while ensuring that a large precursor *m/z* range can be covered.

#### Setting the collision energy in SWATH‐MS

Whereas for DDA, SRM and PRM acquisition methods, the collision energy can be set individually on a per‐precursor basis (based on precursor *m/z* value and charge state), or even optimized for a specific transition (SRM), the collision energy in SWATH‐MS can only be set on a per‐isolation window basis. In other words, if two peptides with different charge states are selected in the same precursor isolation window, they will be fragmented with the same collision energy, which is likely to be suboptimal for at least one of the charge states. However, this suboptimal peptide fragmentation can be compensated for, to some degree, as long as the measured relative fragment ion intensities are similar to those in the spectral library used to query the data. This can be achieved by acquiring DDA fragment ion spectra for spectral library generation with a charge‐state‐independent collision energy equation, i.e. a collision energy varying with the precursor *m/z* but identical for all charge states. Note that this strategy may come at the cost of lost identifications in the DDA analysis due to suboptimal fragmentation of higher charge state peptides. This suboptimal fragmentation might be partially rescued by ramping the collision energy within a SWATH window, for example from −15 to +15 V, over the course of MS2 accumulation in both DDA and SWATH‐MS mode.

In summary, for Q‐TOF instruments, we recommend to set a collision energy proportional to the *m/z* of a 2+ charged precursor for DDA library generation and to apply the same collision energy of a theoretical 2+ precursor located in the centre of the precursor isolation window for SWATH‐MS. Further, we recommend to systematically assess the fragmentation similarity between instruments and external public spectral libraries. Software tools that support such comparative analysis are readily available (Toprak *et al*, 2014). If significant differences between the fragmentation pattern of a spectral library and a SWATH‐MS measurement are identified, it might be worth to optimize the used collision energy equation in the SWATH‐MS measurements until a maximal similarity to the spectral library is found. This will ensure optimal portability of the peptide query parameters across instruments and laboratories.

#### Should adjacent precursor isolation windows be overlapping?

**Figure 4:**
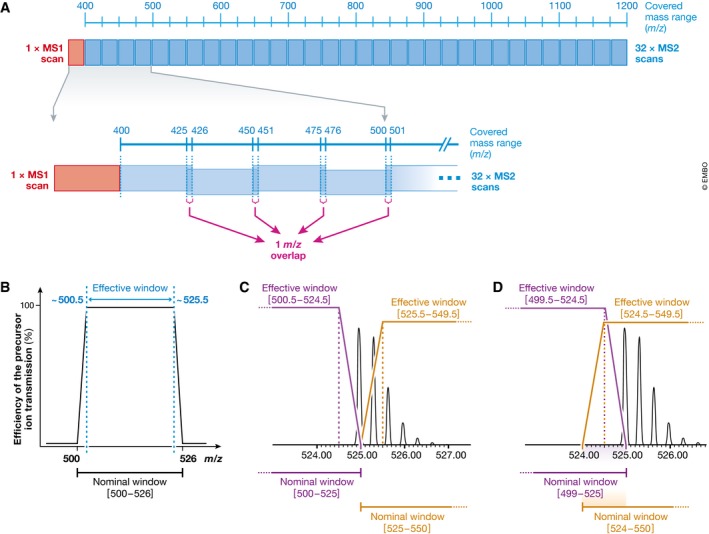
The rationale for using slightly overlapping precursor isolation windows (A) In the first implementation of SWATH‐MS, a 1 *m/z* overlap between adjacent SWATH windows was used to compensate the inefficient ion transmission of the quadrupole at the edges of the precursor isolation window (B) and to limit the effect of precursor isotope splitting between windows (C and D). (B) Schematic representation of the efficiency of ion transmission with a state‐of‐the‐art quadrupole mass analyser filtering for the [500–526] *m/z* range. (C) Theoretical isotopic distribution of the doubly charged peptide MLSYPITIGSLLHK (*m/z* = 524.965). If non‐overlapping nominal windows are used [(500–525) and (525–550)], the isotopic profile is split between both windows and falls within the inefficient ion transmission range of the quadrupole [effective windows from ca. (500.5–524.5) and (525.5–549.5)]. Even if the window edge is placed at a mass where no precursor mass is supposed to occur (for example 525.1), the issue of inefficient ion transmission and loss of parts of the isotopic envelope would remain. (D) With overlapping nominal windows [(499–525) and (524–550)], most of the precursor isotopic pattern will be transmitted within the effective ion transmission range of the quadrupole [effective windows from ca. (499.5–524.5) and (524.5–549.5)].

In the original SWATH‐MS publication, we suggested to acquire consecutive SWATH windows with a 1 *m/z* precursor isolation *m/z* overlap (Fig 4A and Table EV1) (Gillet *et al*, 2012). This was implemented for two reasons: first, precursor ion isolation of the quadrupole mass analyser does not work with 100% efficiency over the whole mass range, but is compromised at the borders of the isolation windows (Fig 4B). Therefore, a small *m/z* overlap on both window edges compensates to some degree signal losses due to not perfectly square‐shaped ion transmission efficiencies and ensures that precursors with *m/z* located at the border of the isolation window suffer as little as possible from signal attrition. Second, overlapping windows ensure maximal transfer of the complete isotopic pattern of precursors that would otherwise be split between two consecutive isolation windows for precursor masses close to a window edge (compare Fig 4C and D). Importantly, unless the entire precursor isotopic envelope gets isolated, the fragment ions will show a distorted isotopic pattern, which will potentially result in lower scoring of this peptide at data analysis stage (see section “Automated peak group scoring”). The SWATH‐MS schemes published to date with 1 *m/z* window overlaps do not completely account for the problems mentioned above in the most extreme cases and, as such, slightly larger overlaps could be considered in future schemes. Note that, even if the precursor isotopic distribution is split between two consecutive isolation windows (as shown in Fig 4C), *label‐free* quantification of that peptide using fragment ion intensities will still be accurate across runs, as long as the window geometries are reproducible and consistent between scans and injections (Egertson *et al*, 2015). A problem may occur, though, in the case of labelled SILAC‐type experiments when one of the precursor isotopic distribution (e.g. the light form) would be split but the heavy is not. In this case, the light‐to‐heavy monoisotopic fragment ion ratios would be distorted due to the fact that part of the intensity of the monoisotopic peak at the fragment ion level is arising from the non‐monoisotopic peaks in the precursor isotopic envelope.

Another possibility to minimize the impact of imperfect ion transmission efficiency is to purposely position the isolation window edges at masses where peptide *m/z* values are unlikely to occur as described by Egertson *et al* (2013). Such theoretically optimal window placement can be realized easily using the Skyline software (Egertson *et al*, 2015). Though this does not *per se* account for the signal attrition at the window borders, nor prevents precursor isotopes to split in different windows, it ensures that the monoisotopic precursor mass does not fall exactly on a window border.

In summary, the 1 *m/z* window overlapping suggested by Gillet *et al* (2012) causes a small loss in terms of selectivity, because the effectively measured windows are slightly broader. However, this is justified by positive effects like minimized signal losses for peptides falling at the sides of the isolation windows and more complete transfer of the isotopic envelope. If no window overlap is implemented, it is highly recommendable to perform optimal window placement as suggested by Egertson *et al* (2013).

#### Advanced DIA acquisition schemes with improved precursor selectivity

**Figure 5:**
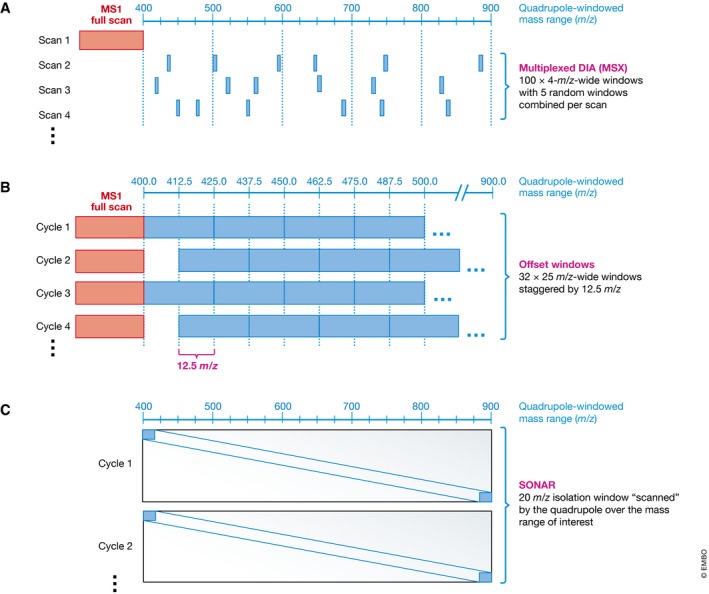
Advanced DIA acquisition schemes with improved precursor selectivity (A) Multiplexed DIA (MSX) (Egertson *et al*, 2013) can be used to improve data selectivity by isolating and co‐fragmenting at each cycle different non‐contiguous precursor mass regions. (B) In the offset‐windowed DIA approach, the precursor isolation window boundaries are offset by a discrete mass between consecutive cycles. For example, 25 *m/z* isolation windows get shifted by ± 12.5 *m/z* in each cycle. (C) In the “scanning quadrupole” isolation approach, termed SONAR (Moseley *et al*, 2018), the instrument continuously scans a wide precursor isolation window through the entire precursor mass range of interest, for example by scanning a mass range from 500 to 900 *m/z* using 200 × 20 *m/z* wide windows swiped with an 2 *m/z* increment.

The selectivity of SWATH‐MS is a direct function of the precursor isolation window width. Further, selectivity directly affects the achievable dynamic range and sensitivity of a SWATH‐MS measurement. Recently, novel acquisition methods have been proposed to improve the selectivity of SWATH‐MS (Fig 5).

##### Multiplexed DIA (MSX)

In 2013, the MacCoss group reported the development of MSX (Egertson *et al*, 2013), a multiplexed DIA scheme. Instead of recording MS2 spectra for precursors selected in systematic SWATH windows of 20 *m/z* width, MSX records at each cycle fragment ions for peptides selected from different multiple *non‐contiguous* isolation windows (e.g. five non‐contiguous isolation windows of 4 *m/z*, Fig 5A). For the MSX setup, the overall absolute selectivity per spectrum is theoretically the same as for a 20 *m/z* wide SWATH‐MS method. However, in MSX, a given peptide precursor will randomly co‐fragment with different species in each cycle, resulting in different interferences over the elution profile of a peptide. In its current implementation, MSX requires an instrument with multiplexed trapping capabilities (ruling out Q‐TOFs).

##### Offset isolation windows intra‐run

In the offset window approach, the precursor isolation window boundaries are shifted back and forth between consecutive cycles in a way that the isolation windows overlap by a significant proportion (e.g. 25 *m/z* isolation windows overlapping by 12.5 *m/z*; Fig 5B). Similarly to MSX, the offset acquisition scheme results in differential precursor ion co‐isolation and co‐fragmentation in every second cycle. Therefore, interferences that originate from species co‐selected in the differently overlapping part of the isolation window can be partitioned away and mathematically removed. This approach shows improved selectivity and lower limit of quantification compared to the standard non‐overlapping approach, without extra cost related to cycle time, accumulation time, covered mass range or resolving power (https://skyline.gs.washington.edu/labkey/files/home/software/Skyline/2013-ASMS-Overlapped-DIA.pdf). However, the method still requires the use of an automated algorithm which pre‐empts easy direct raw data inspection or direct raw fragment ion extraction prior to data deconvolution. Unlike MSX, this method is also possible on non‐trapping instruments such as Q‐TOFs.

##### Scanning quadrupole isolation

The latest advancement of SWATH‐MS was recently published under the name “SONAR” (Waters) by Moseley *et al* (2018). Instead of “stepping” through consecutive isolation windows cyclically one after the other, the quadrupole constantly “scans”, for example, a 24 *m/z* wide isolation window through the entire precursor mass range of interest (Fig 5C). In other words, while with the original SWATH‐MS method, a given precursor will conceptually be present in only one isolation window and absent from all the others, with the “scanning windows” one can see fragment ion signals appearing and disappearing accordingly to the precursor's isotopes entrance and exit from the scanning window. This essentially adds a fourth dimension on top of retention time, mass and intensity to the SWATH‐MS data. The method will require significant modifications of the existing peptide‐centric analysis tools, but intuitively, this extra dimension will allow to access another level of selectivity.

## Peptide‐centric scoring of SWATH‐MS data

**Figure 6:**
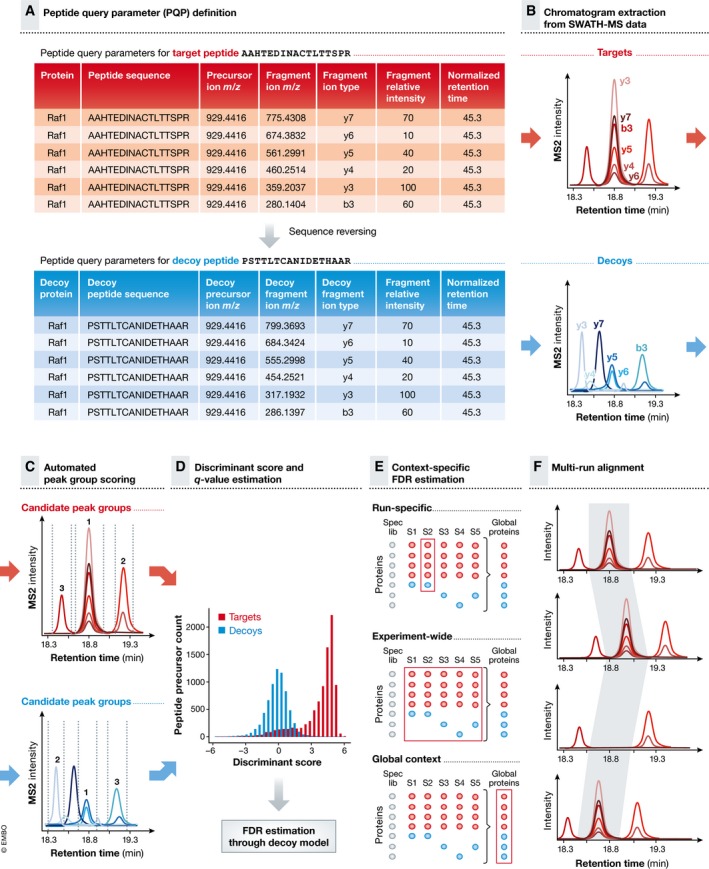
Principle of peptide‐centric scoring of SWATH‐MS data (A) Peptide‐centric scoring begins with a set of peptide query parameters (PQPs), which represent retention time, precursor ion masses, fragment ion masses and fragment ion signal intensity coordinates for the target peptides (red table). PQPs are also required for decoy peptides and are generated, for example, by reversing the amino acid sequence of target peptides, while keeping the terminal amino acid (blue table). Decoy peptides are used to assess the chance that peptides which are expected to be absent in the sample may also be detected by chance. (B) Extracted ion chromatograms (XICs) are generated based on PQPs from the continuously acquired SWATH‐MS2 spectra for target and decoy peptides. This results in a transformed and reduced data structure similar to data generated by targeted proteomics (SRM or PRM). (C) Fragment ion chromatograms are grouped according to their peptide association and “peak groups” with defined peak boundaries in the retention time dimension are selected. (D) For both target and decoy peak groups, a range of chromatogram‐ and spectrum‐based scores are computed and combined to a discriminant score by a semi‐supervised learning approach. The false discovery rate (FDR) of a set of detected peptides can be estimated by statistical modelling of the score distributions of target and decoy peptides. (E) For large‐scale SWATH‐MS analyses, error rate control should not only be performed on the peptide level, but should be extended to the protein level. Further, in large experiments including many samples, it might not be sufficient to conduct error rate control individually per run (“run‐specific” context), but better on an “experiment‐wide” scale. The “global” context considers only the best scoring detected peak groups, peptides or inferred proteins over all runs. (F) A multi‐run alignment allows to correct or reinforce confidence in peak detection by leveraging the chromatographic time consistency and transfer of detection confidence across runs.

Over the past 20 years, a wide variety of strategies have been developed to analyse DIA data. These strategies can be classified into two groups, depending on whether they are based on querying the data in a spectrum‐centric or in a peptide‐centric manner (Ting *et al*, 2015). In this tutorial, we focus on the chromatogram‐based peptide‐centric scoring analysis workflow for SWATH‐MS data illustrated in Fig 6 and discuss specific considerations for each step. For alternative analysis strategies, we refer to recent reviews (Chapman *et al*, 2014; Bilbao *et al*, 2015).

Several software tools have been developed that support all or most steps of the workflow shown in Fig 6. These include for example OpenSWATH (Rost *et al*, 2014) (part of OpenMS, Rost *et al*, 2016b; Sturm *et al*, 2008), Skyline (MacLean *et al*, 2010), PeakView (SCIEX) and Spectronaut (Bruderer *et al*, 2015) (Biognosys). A recent comparative study (Navarro *et al*, 2016) using a ground truth data set has demonstrated remarkable agreement for the qualitative and quantitative results produced by those peptide‐centric scoring tools as well as by the spectrum‐centric scoring algorithm DIA‐Umpire (Tsou *et al*, 2015) that was also included in the study.

### Peptide query parameter (PQP) definition

The first step in a peptide‐centric analysis is the definition of PQPs. While recently developed algorithms can score data without the need for empirically derived PQPs (Tsou *et al*, 2015; Ting *et al*, 2017), and thereby make the availability of prior knowledge in form of spectral libraries obsolete, empirical PQPs derived from experimental fragment ion spectra can still provide higher sensitivity, especially when working with rather large precursor isolation windows (> 5 *m/z*) during data acquisition. Depending on the sample complexity, several high‐quality peak groups can be found for a given queried peptide. To quantitatively describe the frequency of this phenomenon, decoy peptides are used, which are expected to closely model the properties of the target peptides in terms of their chromatographic and mass spectrometric parameter space, but which should not be present in the sample. The decoy PQPs are generated *in silico* from the target peptide list by reversing or shuffling the amino acid sequence (Fig 6A). Decoy peptides are crucial for controlling the error rate of peptide detection and protein inference in SWATH‐MS data, because they can be used to estimate the effect of random co‐elution of fragment ion chromatograms in the underlying data (Rost *et al*, 2014). It is generally recommended to use the same number of fragment ions per peptide for both targets and decoys to not introduce any biases during peak scoring, because peptide queries with six co‐eluting fragment ions can have different score distributions than queries with only three fragment ions.

### Chromatogram extraction

The next step of a peptide‐centric data analysis strategy is to use the defined PQPs to extract precursor and fragment ion chromatograms for the peptides of interest. As a peptide elutes from the column, the signal intensity of its queried fragment ions will vary synchronously following a usually Gaussian‐like peptide elution profile, forming a chromatographic “peak group” that will be used to assess the peptide detection and to estimate its quantity (Fig 6B). Fragment ion chromatograms are extracted in the same manner for the target and decoy peptides. In essence, this chromatogram extraction step transforms, and thus reduces the multiplexed SWATH‐MS2 spectra, to a data structure highly similar to SRM or PRM data.

To substantially improve the selectivity of peptide‐centric SWATH‐MS data analysis, the chromatogram extraction is usually not conducted throughout the whole chromatographic gradient length but only in a retention time window centred around the expected elution time for the queried peptide (similar to the scheduled SRM strategy). A set of shared endogenous (Parker *et al*, 2015b; Bruderer *et al*, 2016) or synthetic spike‐in peptides (Escher *et al*, 2012) can be used to mediate transformation of the previously empirically determined elution times and to restrict the size of retention time extraction window as much as possible. The specific elution times of such reference peptides serve as beacons to define a normalized retention time (such as in the iRT approach; Escher *et al*, 2012) to which the elution times of query peptides can be aligned. The functionality to automatically perform a normalized indexed retention time alignment is implemented in many popular SWATH‐MS data analysis tools (e.g. OpenSWATH, Skyline, PeakView and Spectronaut).

The mass tolerance or width of the ion extraction also directly impacts the selectivity of the chromatographic trace signals and thus the derived identification scores and abundance estimates. In general, an extraction width of half of the mass spectrometric peak width provides a good compromise between maximising the fraction of recovered ion signals while maintaining a reasonable selectivity of the extracted data. In practice, we suggest applying an extraction width of ~50 ppm or less for MS2 measurements performed with a resolution of 15,000.

Chromatographic extraction can be performed on profile (raw) or centroided MS2 data level. Centroided MS2 data are significantly smaller in size and were shown to perform similarly to profile data in terms of identification depth and quantification accuracy (Navarro *et al*, 2016). The optimal extraction window width for centroided data may be less than that of the same data extracted in profile mode, because in this case the extraction window width can be optimized with respect to the mass error distribution. However special attention should be given to the choice of centroiding algorithm used. We have observed that they can dramatically affect the data in terms of mass accuracy, signal‐to‐noise levels and relative fragment ion intensity (e.g. depending on whether the centroiding is done on peak height or peak area) (Toprak *et al*, 2014). Accurate centroiding is especially challenging for SWATH‐MS data due to the high level of co‐fragmenting peptides and the high level of overlapping fragment ion signals in the MS2 spectra. Such algorithm‐specific artefacts will affect the scoring of the fragment ion peak groups as well as the statistics of peptide detection. It is thus recommended to follow the instrument vendor and algorithm developer instructions for the choice of centroiding algorithm.

Finally, if the SWATH‐MS data acquisition scheme includes MS1 scans within a chromatography‐compatible cycle time, precursor ion chromatograms can also be extracted from the MS1 data and scored in combination with the fragment ion peak group to enhance confidence in the peptide detection.

### Automated peak group scoring

In the next step of the workflow, an algorithm defines the left and right borders of one or several potential chromatographic peak groups and scores each of these candidate peptide signals individually (Fig 6C). This is done independently for the extracted target as well as decoy chromatographic traces. During this scoring process, a variety of individual scores is computed (Reiter *et al*, 2011). Those can be categorized into five types: (i) scores related to chromatographic performance, (ii) scores correlating the measured data to the information from the spectral library, (iii) scores correlating the data to external or internal isotope‐labelled standards (if available), (iv) scores taking into account the high resolution and accurate mass of the MS2 scans and (v) scores that exploit information from the precursor ion (MS1) scans. All or most of those individual scores have been implemented in one way or another into the SWATH‐MS software tools OpenSWATH, Skyline, PeakView and Spectronaut, which all automatically combine them into a final discriminant score.

### Discriminant score and q‐value estimation

By applying the individual scores described before to extracted ion chromatograms of target and decoy peptides from SWATH‐MS data, individual score distributions can be computed. Ideally, in these distributions, “true positive” target and “false positive” decoy peptides are clearly separable. However, depending on experiment‐specific factors such as sample complexity, instrument performance and SWATH‐MS method setup, some scores might perform better than others, while a single individual score is generally not discriminative enough to ascertain the detection and correct quantification of a given peptide. Therefore, it is beneficial to combine the individual scores into a single discriminant score in a way that allows the most sensitive recall of the peptide queries (Fig 6D). This goal can be achieved by using a semi‐supervised learning algorithm (Kall *et al*, 2007) that converges iteratively towards the most optimal set of weighted individual score combinations to separate targets and decoys. The peak group with the highest discriminant score (rank 1, Fig 6C) is then usually considered as the most likely detected peak group for a set of queried peptides, which may eventually be revised upon across‐run alignment.

Several requirements need to be fulfilled in order to allow successful learning and proper statistical modelling of SWATH‐MS data. First, a sufficient number of high scoring true positive peptides are necessary for the semi‐supervised algorithm to learn the scoring characteristics of true detection events and to achieve a sensible combination of individual weight‐feature scores. Second, the decoy peptides must appropriately represent peptides that are not detectable in the sample.

In general, we highly recommend to inspect target and decoy score distribution plots that most SWATH‐MS analysis tools report to verify that (i) assumptions made with regard to the decoy distributions (e.g. normality) are not violated, (ii) the target scores appear approximately as a bimodal distribution when analysing complex samples with medium to large spectral libraries and (iii) the decoy distribution matches the true‐negative part of the bimodal target distribution both in terms of apex, shape and width (Fig 6D). In scenarios, where the spectral library overlaps to a very high degree (> 90%) with the detected precursor ions of the sample, the true‐negative target distribution might be very small, thus rendering criterion (iii) difficult to judge (Reiter *et al*, 2011). If one or more of those conditions are not fulfilled, it is likely that either the machine learning or the q‐value estimation step has failed or are biased, which could result in misleading statistics and inappropriate error rate control.

### Context‐specific error rate estimation

The above described strategies are commonly applied to individual runs separately. However, under certain circumstances, this might introduce biases, especially when large numbers of peptides are queried across data sets consisting of a large number of heterogeneous samples, or when spectral libraries are used that were assembled from different sample types (e.g. obtained from public repositories) or contain a large number of true‐negative peptides and proteins for the tested samples. We therefore suggest to adapt the analysis strategy according to requirements of the specific data set as described recently by Rosenberger *et al* (2017a). Briefly, to ensure comparable scoring and statistics throughout the complete data set, the semi‐supervised machine learning step should be conducted in an experiment‐wide fashion on all the candidate peak groups for all runs at once (Fig 6E). For practical reasons (file size and memory restrictions), this step can also be performed on a representative selection of peptide queries randomly subsampled through each sample of the data set. Then, the same score weights are applied to all the candidate peak groups throughout all the runs of the data set (Rosenberger *et al*, 2017a).

While a 1% peptide FDR threshold on a per run basis (referred to as “run‐specific” context) might be sufficient for small sample sizes analysed with sample‐specific libraries, accumulation of false positives will occur when conducting large numbers of queries across large sample sets. Similar as for DDA database search strategies, more stringent score cut‐offs become necessary on large data sets to account for multiple hypothesis testing. We therefore recommend to also use target and decoy score distributions to conduct error rate control at the protein level in addition to the peptide level (Rosenberger *et al*, 2017a). Further, we suggest assessing the FDR in an “experiment‐wide” context where q‐values are estimated over all samples using a single score distribution, as well as in a “global” context, where only the best scoring instance of a given peptide or inferred protein from all samples in the experiment is used. We recommend that the list of peptide queries and inferred proteins identified in the “global” context at 1% FDR can be used to filter the resulting data from the “experiment‐wide” context to prevent accumulation of false positives in experiments with many samples (Fig 6E; Rosenberger *et al*, 2017a). The above described context‐specific error estimation has been implemented in PyProphet (Rosenberger *et al*, 2017a), a component of the OpenSWATH workflow, and Spectronaut (Bruderer *et al*, 2017).

### Across‐run alignment and transfer of identification confidence

A main assumption of peptide‐centric scoring is that only the best scoring peak group per peptide represents the true peptide signal. This may however not always be the case, for example when the peptide abundance drops below the limit of detection in one or several runs of a sample cohort and a candidate peak group that does not originate from the target peptide achieves a better score. Frequently, this wrong peak group is detected at a different retention time than the true peptide, resulting in a false value in the final peptide quantification matrix. By re‐emphasizing the consistency of the retention time information across samples and aligning the peak boundaries of the peak group from the run where the peptide was most confidently identified, it is possible to detect and amend those false peak group ranking mistakes (Fig 6F). This process sometimes re‐ranks the peak groups or may rescue some peak groups at the expected retention time that would otherwise not have passed the 1% peptide FDR threshold in those specific runs. An algorithm for multi‐run retention time alignment is TRIC (TRansfer of Identification Confidence), a component of the OpenSWATH workflow (Rost *et al*, 2016a).

In general, multi‐run alignment is similar to what has already been developed and applied successfully for the analysis of DDA data (Prakash *et al*, 2006; Mueller *et al*, 2007; Cox *et al*, 2014). However, while in DDA peptide identification and quantification are often inferred across runs based solely on retention time and precursor *m/z*, in SWATH‐MS the full information of all fragment ions can be used as part of the alignment strategy and to control the error rate.

### Peptide and protein quantification

Just like any other bottom‐up LC‐MS/MS proteomic methods, SWATH‐MS provides quantification data on peptide level and peptide quantities are typically computed by summing or averaging the integrated peak area of several fragment ions. It is not necessary to use all of the queried fragment ions per peptide to perform accurate quantification. Rather only high‐quality and interference‐free fragment traces should be selected and for such a task automated algorithms have been developed (Keller *et al*, 2015; Teleman *et al*, 2015). Additionally, MS1‐based quantification on the precursor extraction ion chromatograms may provide orthogonal quantification information (Rardin *et al*, 2015). In many instances, however, the lower limit of quantification (LLOQ) appears to be more strongly impaired at the precursor than at the fragment ion level (Gillet *et al*, 2012; Egertson *et al*, 2013; Collins *et al*, 2017). This is due to the lower selectivity and the higher dynamic range of detection required in full MS1 scans compared to that of windowed‐isolation MS2 scans.

To infer the abundance of a protein from a SWATH‐MS measurement, the measured intensities of one or many peptides need to be aggregated into a final protein intensity value per sample. For this purpose, various strategies have been developed, for example (i) summing or averaging the n‐most intense peptides per protein (Silva *et al*, 2006; Ludwig *et al*, 2012), (ii) summing up all peptide intensities per sample, regardless of whether they occur across samples or not (iBAQ; Schwanhausser *et al*, 2011), or (iii) considering only those peptides per protein that occur in the two samples to be compared (LFQ; Cox *et al*, 2014). Optionally, those aggregated protein intensity values can be further normalized by a factor representative of the detectability of that protein, for example protein length (Zybailov *et al*, 2006), number of theoretical peptides generated (Schwanhausser *et al*, 2011) or a computed peptide detection probability score (Lu *et al*, 2007). Missing values can be either recovered using the re‐quantification values provided from the across‐run re‐alignment analysis (Rost *et al*, 2016a) or using statistical imputation as provided by other tools (Karpievitch *et al*, 2012). Compared to SRM or PRM, which commonly only use a few measured targeted peptides for protein inference, and to DDA, which may not identify and quantify the same peptides per protein through large sample sets, SWATH‐MS peptide‐centric analysis intrinsically offers a more consistent and complete choice in peptides for protein quantification.

Several statistical tools have been developed to assess differential peptide or protein abundance between experimental groups and to compute the relative fold change in peptide or protein quantification starting from fragment ion level data (Chang *et al*, 2012). For most consistent relative protein quantification comparisons, we recommend to systematically use the same, and throughout the complete SWATH‐MS data set the most robust, fragments and peptides per protein. For the selection of these fragments and peptides, information such as conservation of the relative fragment ion signal intensities per peptide and the relative peptide intensities per protein can be leveraged across the whole data set, as MSstats (Choi *et al*, 2014) or mapDIA (Teo *et al*, 2015) do for fold‐change statistics computation.

### Post‐translational modifications

SWATH‐MS peptide‐centric scoring is directly compatible with measuring peptides carrying post‐translational modifications (PTMs). However, analysis of modified peptides by SWATH‐MS presents additional challenges that stem from the often high similarity of MS2 spectra arising from related peptide species, including the non‐modified peptide, modified peptides with the same backbone carrying the same modification at different sites or peptides with the same backbone carrying (near) isobaric modifications. Such peptides may be co‐isolated in the same SWATH‐MS window and produce multiple high scoring peak groups.

As in DDA analysis, specific strategies have been developed to confidently identify or detect modified peptides in DIA and SWATH‐MS data sets. The most straightforward way is to directly use PQPs of the modified peptide versions, for example from spectral libraries that were acquired from DDA analyses of PTM‐enriched samples or with open modification search strategies (Na & Paek, 2015). An alternative strategy relies on using non‐modified PQPs to query for modified peptides and focuses on additional high scoring peak groups. Those additional peak groups may be detected in the same isolation window as the non‐modified peptides or in other isolation windows. Such strategies have been implemented in MSPLIT‐DIA (Wang *et al*, 2015) and SWATHProphet^PTM^ (Keller *et al*, 2016) and were shown to work most successfully when the peptide fragmentation shows enough similarity up to the site of the modification (Toprak *et al*, 2014).

A recently introduced algorithm termed “Inference of PeptidoForms” (IPF) (Rosenberger *et al*, 2017b) extends the standard OpenSWATH workflow to modified peptides. IPF uses a two‐stage workflow in which the first pass resembles a standard OpenSWATH analysis whereby typically, about six fragment ions from DDA‐ or DIA‐derived spectral libraries are extracted and scored to detect high‐quality peak groups. These transitions need to be specific for the targeted peptide sequence and types and numbers of modifications; however, they do not need to be specific for positional isomers. In a second step, IPF uses XICs extracted both for precursor signals in MS1 and theoretically predicted fragment ions that can differentiate positional isomers. This information is then integrated using a Bayesian hierarchical model leading to a single peptidoform confidence score for each detected peak group. The site localization is thus independently reassessed, even if the underlying peptidoform is not present in the spectral library.

In summary, PTM‐SWATH analyses are applicable to total cellular lysates as well as to samples enriched for specific types of modifications, such as phospho‐enriched samples. In recent years, several different PTM‐types have been successfully studied with SWATH‐MS (Krautkramer *et al*, 2015; Sidoli *et al*, 2015; Lawrence *et al*, 2016; Rosenberger *et al*, 2017b). Particularly in experiments involving many experimental conditions or replicates, similar improvements in terms of consistent detection and quantification can be expected for modified and unmodified peptides.

## Outlook

Since the first implementations of DIA in the early 2000s (Masselon *et al*, 2000; Purvine *et al*, 2003; Venable *et al*, 2004), numerous improvements in terms of data acquisition speed, mass accuracy and resolution have taken place on the instrument side. Also, since the first description of a peptide‐centric data analysis workflow (Gillet *et al*, 2012), substantial progress has been achieved in terms of spectral library generation (Schubert *et al*, 2015a), automated data analysis pipelines (Rost *et al*, 2014, 2016a; Keller *et al*, 2015) and statistical control in peptide and protein error rates (Rosenberger *et al*, 2017a). The excellent reproducibility and accuracy of SWATH‐MS data acquisition have been proven in a large study comparing data acquired in different laboratories worldwide (Collins *et al*, 2017). Also, the robustness of peptide‐centric data analysis tools has been demonstrated recently by comparing five major software tools currently used for peptide‐centric scoring of SWATH‐MS data (Navarro *et al*, 2016). The development of SWATH‐MS laid the foundation for novel large‐scale biological studies, such as the study of mitochondrial links to liver metabolism in a mouse reference population (Williams *et al*, 2016), the study of phosphoproteomic and chromatin signature in response to drug treatments in cancer cell lines (preprint: Litichevskiy *et al*, 2018) or the contribution of heritability and environment to different traits in pairs of monozygotic and dizygotic twins (Liu *et al*, 2015). Throughout this tutorial, we have documented the advancements and referenced established workflows, software tools and analysis pipelines that will hopefully support and facilitate SWATH‐MS experiments by a large user community in the years to come.

Although most published SWATH‐MS studies to date used spectral libraries generated by DDA, novel approaches that do not rely on libraries were developed. Spectrum‐centric scoring approaches such as DIA‐Umpire (Tsou *et al*, 2015) or Group‐DIA (Li *et al*, 2015) generate pseudo‐MS2 spectra directly from DIA data and subject them to conventional database search algorithms. Peptide‐centric library‐free analysis tools such as FT‐ARM (Weisbrod *et al*, 2012) or PECAN (Ting *et al*, 2017) use theoretical fragment ion predictions to query and score the multiplexed MS2 spectra of SWATH‐MS data. Interestingly, tools that rely on prior knowledge in the form of spectral libraries seem to deal better with lower selectivity data than library‐free tools (Navarro *et al*, 2016; Ting *et al*, 2017). This finding suggests that, as measurement selectivity improves with better instruments (smaller precursor isolation windows, higher peak capacity chromatography, scanning quadrupoles, ion mobility separations, etc.), prior knowledge for data analysis might become less important. On the other hand, prior knowledge in the form of organism‐wide deep proteome data sets from endogenous samples or synthetic proteomics is likely to increase substantially during the same time period. This might spur the development of novel analysis methods that can better leverage such libraries.

In the future, we expect that mass spectrometers will continue to improve in sensitivity and scanning speed. Hence, it is foreseeable that the time necessary to acquire high‐quality precursor and fragment ion spectra will decrease, allowing for higher number of narrower isolation windows and enabling to approach the 1–3 *m/z* isolation width commonly used during DDA acquisition and PRM. We speculate that increasing instrument sensitivity and speed will reach a threshold where ultimately the benefits of all currently employed data acquisition methods can be achieved with a single “super” method. The question then will no longer be whether one should do a DDA, DIA or targeted experiment, but whether to use a peptide‐centric or spectrum‐centric data analysis strategy. It seems unlikely that a purely spectrum‐centric approach, where each consecutive MS2 scan is subjected to a database search algorithm independently, would be most effective, because the rich information in the chromatographic dimension and prior information about the fragmentation pattern would not be leveraged. As such, it seems more likely that either peptide‐centric approaches as described in detail in this tutorial or hybrid methods between spectrum‐ and peptide‐centric analyses best exploit the highly comprehensive and selective data sets in the future.

Other future‐oriented features of DIA in general, and SWATH‐MS in particular, are the simplicity of the underlying data acquisition method and the suitability for high‐throughput proteomics. Already today ~50% of the MS‐detectable proteome can be reproducibly measured by SWATH‐MS at a relatively fast time scale (< 1 h per sample). This progress will hopefully allow to further democratize access to proteomics data in the future and to perform novel types of cross study comparisons, which are goals that the proteomics community had difficulties achieving in the past.

## Conflict of interest

The authors declare that they have no conflict of interest. RA is a shareholder in the company Biognosys which operates in the field of research covered by this article.

## Supporting information



Appendix

Table EV1

## References

[msb178126-bib-0001] Aebersold R , Mann M (2016) Mass‐spectrometric exploration of proteome structure and function. Nature 537: 347–355 27629641 10.1038/nature19949

[msb178126-bib-0002] Ahrne E , Glatter T , Vigano C , Schubert C , Nigg EA , Schmidt A (2016) Evaluation and improvement of quantification accuracy in isobaric mass tag‐based protein quantification experiments. J Proteome Res 15: 2537–2547 27345528 10.1021/acs.jproteome.6b00066

[msb178126-bib-0003] Bereman MS , Beri J , Sharma V , Nathe C , Eckels J , MacLean B , MacCoss MJ (2016) An automated pipeline to monitor system performance in liquid chromatography‐tandem mass spectrometry proteomic experiments. J Proteome Res 15: 4763–4769 27700092 10.1021/acs.jproteome.6b00744 PMC5406750

[msb178126-bib-0004] Bilbao A , Varesio E , Luban J , Strambio‐De‐Castillia C , Hopfgartner G , Muller M , Lisacek F (2015) Processing strategies and software solutions for data‐independent acquisition in mass spectrometry. Proteomics 15: 964–980 25430050 10.1002/pmic.201400323

[msb178126-bib-0005] Blainey P , Krzywinski M , Altman N (2014) Points of significance: replication. Nat Methods 11: 879–880 25317452 10.1038/nmeth.3091

[msb178126-bib-0006] Bruderer R , Bernhardt OM , Gandhi T , Miladinovic SM , Cheng LY , Messner S , Ehrenberger T , Zanotelli V , Butscheid Y , Escher C , Vitek O , Rinner O , Reiter L (2015) Extending the limits of quantitative proteome profiling with data‐independent acquisition and application to acetaminophen‐treated three‐dimensional liver microtissues. Mol Cell Proteomics 14: 1400–1410 25724911 10.1074/mcp.M114.044305 PMC4424408

[msb178126-bib-0007] Bruderer R , Bernhardt O , Gandhi T , Reiter L (2016) High precision iRT retention time prediction in the targeted analysis of data‐independent acquisition and its impact on identification and quantitation. Proteomics 16: 2246–2256 27213465 10.1002/pmic.201500488 PMC5094550

[msb178126-bib-0008] Bruderer R , Bernhardt OM , Gandhi T , Xuan Y , Sondermann J , Schmidt M , Gomez‐Varela D , Reiter L (2017) Optimization of experimental parameters in data‐independent mass spectrometry significantly increases depth and reproducibility of results. Mol Cell Proteomics 16: 2296–2309 29070702 10.1074/mcp.RA117.000314 PMC5724188

[msb178126-bib-0009] Carr SA , Abbatiello SE , Ackermann BL , Borchers C , Domon B , Deutsch EW , Grant RP , Hoofnagle AN , Huttenhain R , Koomen JM , Liebler DC , Liu T , MacLean B , Mani DR , Mansfield E , Neubert H , Paulovich AG , Reiter L , Vitek O , Aebersold R *et al* (2014) Targeted peptide measurements in biology and medicine: best practices for mass spectrometry‐based assay development using a fit‐for‐purpose approach. Mol Cell Proteomics 13: 907–917 24443746 10.1074/mcp.M113.036095 PMC3945918

[msb178126-bib-0010] Chang CY , Picotti P , Huttenhain R , Heinzelmann‐Schwarz V , Jovanovic M , Aebersold R , Vitek O (2012) Protein significance analysis in selected reaction monitoring (SRM) measurements. Mol Cell Proteomics 11: M111.014662 10.1074/mcp.M111.014662 PMC3322573 22190732

[msb178126-bib-0011] Chapman JD , Goodlett DR , Masselon CD (2014) Multiplexed and data‐independent tandem mass spectrometry for global proteome profiling. Mass Spectrom Rev 33: 452–470 24281846 10.1002/mas.21400

[msb178126-bib-0012] Chick JM , Munger SC , Simecek P , Huttlin EL , Choi K , Gatti DM , Raghupathy N , Svenson KL , Churchill GA , Gygi SP (2016) Defining the consequences of genetic variation on a proteome–wide scale. Nature 534: 500–505 27309819 10.1038/nature18270 PMC5292866

[msb178126-bib-0013] Chiva C , Olivella R , Borras E , Espadas G , Pastor O , Sole A , Sabido E (2018) QCloud: a cloud‐based quality control system for mass spectrometry‐based proteomics laboratories. PLoS ONE 13: e0189209 29324744 10.1371/journal.pone.0189209 PMC5764250

[msb178126-bib-0014] Choi M , Chang CY , Clough T , Broudy D , Killeen T , MacLean B , Vitek O (2014) MSstats: an R package for statistical analysis of quantitative mass spectrometry‐based proteomic experiments. Bioinformatics 30: 2524–2526 24794931 10.1093/bioinformatics/btu305

[msb178126-bib-0015] Collins BC , Gillet LC , Rosenberger G , Rost HL , Vichalkovski A , Gstaiger M , Aebersold R (2013) Quantifying protein interaction dynamics by SWATH mass spectrometry: application to the 14‐3‐3 system. Nat Methods 10: 1246–1253 24162925 10.1038/nmeth.2703

[msb178126-bib-0016] Collins BC , Hunter CL , Liu Y , Schilling B , Rosenberger G , Bader SL , Chan DW , Gibson BW , Gingras AC , Held JM , Hirayama‐Kurogi M , Hou G , Krisp C , Larsen B , Lin L , Liu S , Molloy MP , Moritz RL , Ohtsuki S , Schlapbach R *et al* (2017) Multi‐laboratory assessment of reproducibility, qualitative and quantitative performance of SWATH‐mass spectrometry. Nat Commun 8: 291 28827567 10.1038/s41467-017-00249-5 PMC5566333

[msb178126-bib-0017] Cox J , Hein MY , Luber CA , Paron I , Nagaraj N , Mann M (2014) Accurate proteome‐wide label‐free quantification by delayed normalization and maximal peptide ratio extraction, termed MaxLFQ. Mol Cell Proteomics 13: 2513–2526 24942700 10.1074/mcp.M113.031591 PMC4159666

[msb178126-bib-0018] Craig R , Cortens JP , Beavis RC (2004) Open source system for analyzing, validating, and storing protein identification data. J Proteome Res 3: 1234–1242 15595733 10.1021/pr049882h

[msb178126-bib-0019] Deutsch EW , Lam H , Aebersold R (2008) PeptideAtlas: a resource for target selection for emerging targeted proteomics workflows. EMBO Rep 9: 429–434 18451766 10.1038/embor.2008.56 PMC2373374

[msb178126-bib-0020] Deutsch EW , Mendoza L , Shteynberg D , Farrah T , Lam H , Tasman N , Sun Z , Nilsson E , Pratt B , Prazen B , Eng JK , Martin DB , Nesvizhskii AI , Aebersold R (2010) A guided tour of the trans‐proteomic pipeline. Proteomics 10: 1150–1159 20101611 10.1002/pmic.200900375 PMC3017125

[msb178126-bib-0021] Egertson JD , Kuehn A , Merrihew GE , Bateman NW , MacLean BX , Ting YS , Canterbury JD , Marsh DM , Kellmann M , Zabrouskov V , Wu CC , MacCoss MJ (2013) Multiplexed MS/MS for improved data‐independent acquisition. Nat Methods 10: 744–746 23793237 10.1038/nmeth.2528 PMC3881977

[msb178126-bib-0022] Egertson JD , MacLean B , Johnson R , Xuan Y , MacCoss MJ (2015) Multiplexed peptide analysis using data‐independent acquisition and skyline. Nat Protoc 10: 887–903 25996789 10.1038/nprot.2015.055 PMC5127711

[msb178126-bib-0023] Escher C , Reiter L , MacLean B , Ossola R , Herzog F , Chilton J , MacCoss MJ , Rinner O (2012) Using iRT, a normalized retention time for more targeted measurement of peptides. Proteomics 12: 1111–1121 22577012 10.1002/pmic.201100463 PMC3918884

[msb178126-bib-0024] Frejno M , Zenezini Chiozzi R , Wilhelm M , Koch H , Zheng R , Klaeger S , Ruprecht B , Meng C , Kramer K , Jarzab A , Heinzlmeir S , Johnstone E , Domingo E , Kerr D , Jesinghaus M , Slotta‐Huspenina J , Weichert W , Knapp S , Feller SM , Kuster B (2017) Pharmacoproteomic characterisation of human colon and rectal cancer. Mol Syst Biol 13: 951 29101300 10.15252/msb.20177701 PMC5731344

[msb178126-bib-0025] Geiger T , Cox J , Mann M (2010) Proteomics on an Orbitrap benchtop mass spectrometer using all‐ion fragmentation. Mol Cell Proteomics 9: 2252–2261 20610777 10.1074/mcp.M110.001537 PMC2953918

[msb178126-bib-0026] Geyer PE , Kulak NA , Pichler G , Holdt LM , Teupser D , Mann M (2016) Plasma proteome profiling to assess human health and disease. Cell Syst 2: 185–195 27135364 10.1016/j.cels.2016.02.015

[msb178126-bib-0027] Gillet LC , Navarro P , Tate S , Rost H , Selevsek N , Reiter L , Bonner R , Aebersold R (2012) Targeted data extraction of the MS/MS spectra generated by data‐independent acquisition: a new concept for consistent and accurate proteome analysis. Mol Cell Proteomics 11: O111.016717 10.1074/mcp.O111.016717 PMC3433915 22261725

[msb178126-bib-0028] de Graaf EL , Altelaar AF , van Breukelen B , Mohammed S , Heck AJ (2011) Improving SRM assay development: a global comparison between triple quadrupole, ion trap, and higher energy CID peptide fragmentation spectra. J Proteome Res 10: 4334–4341 21726076 10.1021/pr200156b

[msb178126-bib-0029] Kall L , Canterbury JD , Weston J , Noble WS , MacCoss MJ (2007) Semi‐supervised learning for peptide identification from shotgun proteomics datasets. Nat Methods 4: 923–925 17952086 10.1038/nmeth1113

[msb178126-bib-0030] Karlsson C , Malmstrom L , Aebersold R , Malmstrom J (2012) Proteome‐wide selected reaction monitoring assays for the human pathogen Streptococcus pyogenes. Nat Commun 3: 1301 23250431 10.1038/ncomms2297 PMC3535367

[msb178126-bib-0031] Karpievitch YV , Dabney AR , Smith RD (2012) Normalization and missing value imputation for label‐free LC‐MS analysis. BMC Bioinformatics 13(Suppl 16): S5 10.1186/1471-2105-13-S16-S5 PMC3489534 23176322

[msb178126-bib-0032] Keam SP , Gulati T , Gamell C , Caramia F , Huang C , Schittenhelm RB , Kleifeld O , Neeson PJ , Haupt Y , Williams SG (2018) Exploring the oncoproteomic response of human prostate cancer to therapeutic radiation using data‐independent acquisition (DIA) mass spectrometry. Prostate 78: 563–575 29520850 10.1002/pros.23500

[msb178126-bib-0033] Keller A , Bader SL , Shteynberg D , Hood L , Moritz RL (2015) Automated validation of results and removal of fragment ion interferences in targeted analysis of data‐independent acquisition mass spectrometry (MS) using SWATHProphet. Mol Cell Proteomics 14: 1411–1418 25713123 10.1074/mcp.O114.044917 PMC4424409

[msb178126-bib-0034] Keller A , Bader SL , Kusebauch U , Shteynberg D , Hood L , Moritz RL (2016) Opening a SWATH window on posttranslational modifications: automated pursuit of modified peptides. Mol Cell Proteomics 15: 1151–1163 26704149 10.1074/mcp.M115.054478 PMC4813695

[msb178126-bib-0035] Kelstrup CD , Bekker‐Jensen DB , Arrey TN , Hogrebe A , Harder A , Olsen JV (2018) Performance evaluation of the Q exactive HF‐X for shotgun proteomics. J Proteome Res 17: 727–738 29183128 10.1021/acs.jproteome.7b00602

[msb178126-bib-0036] Kong AT , Leprevost FV , Avtonomov DM , Mellacheruvu D , Nesvizhskii AI (2017) MSFragger: ultrafast and comprehensive peptide identification in mass spectrometry‐based proteomics. Nat Methods 14: 513–520 28394336 10.1038/nmeth.4256 PMC5409104

[msb178126-bib-0037] Krautkramer KA , Reiter L , Denu JM , Dowell JA (2015) Quantification of SAHA‐dependent changes in histone modifications using data‐independent acquisition mass spectrometry. J Proteome Res 14: 3252–3262 26120868 10.1021/acs.jproteome.5b00245 PMC4564294

[msb178126-bib-0038] Krzywinski M , Altman N (2013) Points of significance: power and sample size. Nat Methods 10: 1139–1140 10.1038/nmeth.2659 24161969

[msb178126-bib-0039] Krzywinski M , Altman N (2014a) Points of significance: analysis of variance and blocking. Nat Methods 11: 699–700 25110779 10.1038/nmeth.3005

[msb178126-bib-0040] Krzywinski M , Altman N (2014b) Points of view: designing comparative experiments. Nat Methods 11: 597–598 25019145 10.1038/nmeth.2974

[msb178126-bib-0041] Kulkarni S , Koller A , Mani KM , Wen R , Alfieri A , Saha S , Wang J , Patel P , Bandeira N , Guha C , Chen EI (2016) Identifying urinary and serum exosome biomarkers for radiation exposure using a data dependent acquisition and SWATH‐MS combined workflow. Int J Radiat Oncol Biol Phys 96: 566–577 27485285 10.1016/j.ijrobp.2016.06.008 PMC5042854

[msb178126-bib-0042] Kusebauch U , Campbell DS , Deutsch EW , Chu CS , Spicer DA , Brusniak MY , Slagel J , Sun Z , Stevens J , Grimes B , Shteynberg D , Hoopmann MR , Blattmann P , Ratushny AV , Rinner O , Picotti P , Carapito C , Huang CY , Kapousouz M , Lam H *et al* (2016) Human SRMAtlas: a resource of targeted assays to quantify the complete human proteome. Cell 166: 766–778 27453469 10.1016/j.cell.2016.06.041 PMC5245710

[msb178126-bib-0043] Kuster B , Schirle M , Mallick P , Aebersold R (2005) Scoring proteomes with proteotypic peptide probes. Nat Rev Mol Cell Biol 6: 577–583 15957003 10.1038/nrm1683

[msb178126-bib-0044] Lambert JP , Ivosev G , Couzens AL , Larsen B , Taipale M , Lin ZY , Zhong Q , Lindquist S , Vidal M , Aebersold R , Pawson T , Bonner R , Tate S , Gingras AC (2013) Mapping differential interactomes by affinity purification coupled with data‐independent mass spectrometry acquisition. Nat Methods 10: 1239–1245 24162924 10.1038/nmeth.2702 PMC3882083

[msb178126-bib-0045] Lange V , Picotti P , Domon B , Aebersold R (2008) Selected reaction monitoring for quantitative proteomics: a tutorial. Mol Syst Biol 4: 222 18854821 10.1038/msb.2008.61 PMC2583086

[msb178126-bib-0046] Lawrence RT , Perez EM , Hernandez D , Miller CP , Haas KM , Irie HY , Lee SI , Blau CA , Villen J (2015) The proteomic landscape of triple‐negative breast cancer. Cell Rep 11: 630–644 25892236 10.1016/j.celrep.2015.03.050 PMC4425736

[msb178126-bib-0047] Lawrence RT , Searle BC , Llovet A , Villen J (2016) Plug‐and‐play analysis of the human phosphoproteome by targeted high‐resolution mass spectrometry. Nat Methods 13: 431–434 27018578 10.1038/nmeth.3811 PMC5915315

[msb178126-bib-0048] Li Y , Zhong CQ , Xu X , Cai S , Wu X , Zhang Y , Chen J , Shi J , Lin S , Han J (2015) Group‐DIA: analyzing multiple data‐independent acquisition mass spectrometry data files. Nat Methods 12: 1105–1106 26436481 10.1038/nmeth.3593

[msb178126-bib-0049] Litichevskiy L , Peckner R , Abelin JG , Asiedu JK , Creech AL , Davis JF , Davison D , Dunning CM , Egertson JD , Egri S , Gould J , Ko T , Johnson SA , Lahr DL , Lam D , Liu Z , Lyons NJ , Lu X , MacLean BX , Mungenast AE *et al* (2018) A library of phosphoproteomic and chromatin signatures for characterizing cellular responses to drug perturbations. Cell Syst 6: 424–443.e7 29655704 10.1016/j.cels.2018.03.012 PMC5951639

[msb178126-bib-0050] Liu Y , Huttenhain R , Surinova S , Gillet LC , Mouritsen J , Brunner R , Navarro P , Aebersold R (2013) Quantitative measurements of N‐linked glycoproteins in human plasma by SWATH‐MS. Proteomics 13: 1247–1256 23322582 10.1002/pmic.201200417

[msb178126-bib-0051] Liu Y , Chen J , Sethi A , Li QK , Chen L , Collins B , Gillet LC , Wollscheid B , Zhang H , Aebersold R (2014) Glycoproteomic analysis of prostate cancer tissues by SWATH mass spectrometry discovers N‐acylethanolamine acid amidase and protein tyrosine kinase 7 as signatures for tumor aggressiveness. Mol Cell Proteomics 13: 1753–1768 24741114 10.1074/mcp.M114.038273 PMC4083113

[msb178126-bib-0052] Liu Y , Buil A , Collins BC , Gillet LC , Blum LC , Cheng LY , Vitek O , Mouritsen J , Lachance G , Spector TD , Dermitzakis ET , Aebersold R (2015) Quantitative variability of 342 plasma proteins in a human twin population. Mol Syst Biol 11: 786 25652787 10.15252/msb.20145728 PMC4358658

[msb178126-bib-0053] Lu P , Vogel C , Wang R , Yao X , Marcotte EM (2007) Absolute protein expression profiling estimates the relative contributions of transcriptional and translational regulation. Nat Biotechnol 25: 117–124 17187058 10.1038/nbt1270

[msb178126-bib-0054] Ludwig C , Claassen M , Schmidt A , Aebersold R (2012) Estimation of absolute protein quantities of unlabeled samples by selected reaction monitoring mass spectrometry. Mol Cell Proteomics 11: M111.013987 10.1074/mcp.M111.013987 PMC3316728 22101334

[msb178126-bib-0055] MacLean B , Tomazela DM , Shulman N , Chambers M , Finney GL , Frewen B , Kern R , Tabb DL , Liebler DC , MacCoss MJ (2010) Skyline: an open source document editor for creating and analyzing targeted proteomics experiments. Bioinformatics 26: 966–968 20147306 10.1093/bioinformatics/btq054 PMC2844992

[msb178126-bib-0056] Mallick P , Schirle M , Chen SS , Flory MR , Lee H , Martin D , Ranish J , Raught B , Schmitt R , Werner T , Kuster B , Aebersold R (2007) Computational prediction of proteotypic peptides for quantitative proteomics. Nat Biotechnol 25: 125–131 17195840 10.1038/nbt1275

[msb178126-bib-0057] Martens L , Hermjakob H , Jones P , Adamski M , Taylor C , States D , Gevaert K , Vandekerckhove J , Apweiler R (2005) PRIDE: the proteomics identifications database. Proteomics 5: 3537–3545 16041671 10.1002/pmic.200401303

[msb178126-bib-0058] Masselon C , Anderson GA , Harkewicz R , Bruce JE , Pasa‐Tolic L , Smith RD (2000) Accurate mass multiplexed tandem mass spectrometry for high‐throughput polypeptide identification from mixtures. Anal Chem 72: 1918–1924 10784162 10.1021/ac991133+

[msb178126-bib-0059] Matsumoto M , Matsuzaki F , Oshikawa K , Goshima N , Mori M , Kawamura Y , Ogawa K , Fukuda E , Nakatsumi H , Natsume T , Fukui K , Horimoto K , Nagashima T , Funayama R , Nakayama K , Nakayama KI (2017) A large‐scale targeted proteomics assay resource based on an *in vitro* human proteome. Nat Methods 14: 251–258 28267743 10.1038/nmeth.4116

[msb178126-bib-0060] Matthews DE , Hayes JM (1976) Systematic errors in gas chromatography‐mass spectrometry isotope ratio measurements. Anal Chem 48: 1375–1382

[msb178126-bib-0061] McAlister GC , Huttlin EL , Haas W , Ting L , Jedrychowski MP , Rogers JC , Kuhn K , Pike I , Grothe RA , Blethrow JD , Gygi SP (2012) Increasing the multiplexing capacity of TMTs using reporter ion isotopologues with isobaric masses. Anal Chem 84: 7469–7478 22880955 10.1021/ac301572t PMC3715028

[msb178126-bib-0062] McAlister GC , Nusinow DP , Jedrychowski MP , Wuhr M , Huttlin EL , Erickson BK , Rad R , Haas W , Gygi SP (2014) MultiNotch MS3 enables accurate, sensitive, and multiplexed detection of differential expression across cancer cell line proteomes. Anal Chem 86: 7150–7158 24927332 10.1021/ac502040v PMC4215866

[msb178126-bib-0063] Moseley MA , Hughes CJ , Juvvadi PR , Soderblom EJ , Lennon S , Perkins SR , Thompson JW , Steinbach WJ , Geromanos SJ , Wildgoose J , Langridge JI , Richardson K , Vissers JPC (2018) Scanning quadrupole data‐independent acquisition, part A: qualitative and quantitative characterization. J Proteome Res 17: 770–779 28901143 10.1021/acs.jproteome.7b00464 PMC12140809

[msb178126-bib-0064] Mueller LN , Rinner O , Schmidt A , Letarte S , Bodenmiller B , Brusniak MY , Vitek O , Aebersold R , Muller M (2007) SuperHirn ‐ a novel tool for high resolution LC‐MS‐based peptide/protein profiling. Proteomics 7: 3470–3480 17726677 10.1002/pmic.200700057

[msb178126-bib-0065] Muller DB , Schubert OT , Rost H , Aebersold R , Vorholt JA (2016) Systems‐level proteomics of two ubiquitous leaf commensals reveals complementary adaptive traits for phyllosphere colonization. Mol Cell Proteomics 15: 3256–3269 27457762 10.1074/mcp.M116.058164 PMC5054348

[msb178126-bib-0066] Muntel J , Xuan Y , Berger ST , Reiter L , Bachur R , Kentsis A , Steen H (2015) Advancing urinary protein biomarker discovery by data‐independent acquisition on a quadrupole‐orbitrap mass spectrometer. J Proteome Res 14: 4752–4762 26423119 10.1021/acs.jproteome.5b00826 PMC4993212

[msb178126-bib-0067] Na S , Paek E (2015) Software eyes for protein post‐translational modifications. Mass Spectrom Rev 34: 133–147 24889695 10.1002/mas.21425

[msb178126-bib-0068] Navarro P , Kuharev J , Gillet LC , Bernhardt OM , MacLean B , Rost HL , Tate SA , Tsou CC , Reiter L , Distler U , Rosenberger G , Perez‐Riverol Y , Nesvizhskii AI , Aebersold R , Tenzer S (2016) A multicenter study benchmarks software tools for label‐free proteome quantification. Nat Biotechnol 34: 1130–1136 27701404 10.1038/nbt.3685 PMC5120688

[msb178126-bib-0069] Norbeck AD , Monroe ME , Adkins JN , Anderson KK , Daly DS , Smith RD (2005) The utility of accurate mass and LC elution time information in the analysis of complex proteomes. J Am Soc Mass Spectrom 16: 1239–1249 15979333 10.1016/j.jasms.2005.05.009 PMC1769320

[msb178126-bib-0070] O'Brien JJ , O'Connell JD , Paulo JA , Thakurta S , Rose CM , Weekes MP , Huttlin EL , Gygi SP (2018) Compositional proteomics: effects of spatial constraints on protein quantification utilizing isobaric tags. J Proteome Res 17: 590–599 29195270 10.1021/acs.jproteome.7b00699 PMC5806995

[msb178126-bib-0071] Okada H , Ebhardt HA , Vonesch SC , Aebersold R , Hafen E (2016) Proteome‐wide association studies identify biochemical modules associated with a wing‐size phenotype in *Drosophila melanogaster* . Nat Commun 7: 12649 27582081 10.1038/ncomms12649 PMC5025782

[msb178126-bib-0072] Ortea I , Rodriguez‐Ariza A , Chicano‐Galvez E , Arenas Vacas MS , Jurado Gamez B (2016) Discovery of potential protein biomarkers of lung adenocarcinoma in bronchoalveolar lavage fluid by SWATH MS data‐independent acquisition and targeted data extraction. J Proteomics 138: 106–114 26917472 10.1016/j.jprot.2016.02.010

[msb178126-bib-0073] Panchaud A , Scherl A , Shaffer SA , von Haller PD , Kulasekara HD , Miller SI , Goodlett DR (2009) Precursor acquisition independent from ion count: how to dive deeper into the proteomics ocean. Anal Chem 81: 6481–6488 19572557 10.1021/ac900888s PMC3086478

[msb178126-bib-0074] Panse C , Trachsel C , Grossmann J , Schlapbach R (2015) specL–an R/Bioconductor package to prepare peptide spectrum matches for use in targeted proteomics. Bioinformatics 31: 2228–2231 25712692 10.1093/bioinformatics/btv105

[msb178126-bib-0075] Parker BL , Yang G , Humphrey SJ , Chaudhuri R , Ma X , Peterman S , James DE (2015a) Targeted phosphoproteomics of insulin signaling using data‐independent acquisition mass spectrometry. Sci Signal 8: rs6 26060331 10.1126/scisignal.aaa3139

[msb178126-bib-0076] Parker SJ , Rost H , Rosenberger G , Collins BC , Malmstrom L , Amodei D , Venkatraman V , Raedschelders K , Van Eyk JE , Aebersold R (2015b) Identification of a set of conserved eukaryotic internal retention time standards for data‐independent acquisition mass spectrometry. Mol Cell Proteomics 14: 2800–2813 26199342 10.1074/mcp.O114.042267 PMC4597153

[msb178126-bib-0077] Picotti P , Lam H , Campbell D , Deutsch EW , Mirzaei H , Ranish J , Domon B , Aebersold R (2008) A database of mass spectrometric assays for the yeast proteome. Nat Methods 5: 913–914 18974732 10.1038/nmeth1108-913 PMC2770732

[msb178126-bib-0078] Picotti P , Rinner O , Stallmach R , Dautel F , Farrah T , Domon B , Wenschuh H , Aebersold R (2010) High‐throughput generation of selected reaction‐monitoring assays for proteins and proteomes. Nat Methods 7: 43–46 19966807 10.1038/nmeth.1408

[msb178126-bib-0079] Picotti P , Clement‐Ziza M , Lam H , Campbell DS , Schmidt A , Deutsch EW , Rost H , Sun Z , Rinner O , Reiter L , Shen Q , Michaelson JJ , Frei A , Alberti S , Kusebauch U , Wollscheid B , Moritz RL , Beyer A , Aebersold R (2013) A complete mass‐spectrometric map of the yeast proteome applied to quantitative trait analysis. Nature 494: 266–270 23334424 10.1038/nature11835 PMC3951219

[msb178126-bib-0080] Prakash A , Mallick P , Whiteaker J , Zhang H , Paulovich A , Flory M , Lee H , Aebersold R , Schwikowski B (2006) Signal maps for mass spectrometry‐based comparative proteomics. Mol Cell Proteomics 5: 423–432 16269421 10.1074/mcp.M500133-MCP200

[msb178126-bib-0081] Purvine S , Eppel JT , Yi EC , Goodlett DR (2003) Shotgun collision‐induced dissociation of peptides using a time of flight mass analyzer. Proteomics 3: 847–850 12833507 10.1002/pmic.200300362

[msb178126-bib-0082] Rardin MJ , Schilling B , Cheng LY , MacLean BX , Sorensen DJ , Sahu AK , MacCoss MJ , Vitek O , Gibson BW (2015) MS1 peptide ion intensity chromatograms in MS2 (SWATH) data independent acquisitions. Improving post acquisition analysis of proteomic experiments. Mol Cell Proteomics 14: 2405–2419 25987414 10.1074/mcp.O115.048181 PMC4563724

[msb178126-bib-0083] Reiter L , Claassen M , Schrimpf SP , Jovanovic M , Schmidt A , Buhmann JM , Hengartner MO , Aebersold R (2009) Protein identification false discovery rates for very large proteomics data sets generated by tandem mass spectrometry. Mol Cell Proteomics 8: 2405–2417 19608599 10.1074/mcp.M900317-MCP200 PMC2773710

[msb178126-bib-0084] Reiter L , Rinner O , Picotti P , Huttenhain R , Beck M , Brusniak MY , Hengartner MO , Aebersold R (2011) mProphet: automated data processing and statistical validation for large‐scale SRM experiments. Nat Methods 8: 430–435 21423193 10.1038/nmeth.1584

[msb178126-bib-0085] Rosenberger G , Koh CC , Guo T , Rost HL , Kouvonen P , Collins BC , Heusel M , Liu Y , Caron E , Vichalkovski A , Faini M , Schubert OT , Faridi P , Ebhardt HA , Matondo M , Lam H , Bader SL , Campbell DS , Deutsch EW , Moritz RL *et al* (2014) A repository of assays to quantify 10,000 human proteins by SWATH‐MS. Sci Data 1: 140031 25977788 10.1038/sdata.2014.31 PMC4322573

[msb178126-bib-0086] Rosenberger G , Bludau I , Schmitt U , Heusel M , Hunter CL , Liu Y , MacCoss MJ , MacLean BX , Nesvizhskii AI , Pedrioli PGA , Reiter L , Rost HL , Tate S , Ting YS , Collins BC , Aebersold R (2017a) Statistical control of peptide and protein error rates in large‐scale targeted data‐independent acquisition analyses. Nat Methods 14: 921–927 28825704 10.1038/nmeth.4398 PMC5581544

[msb178126-bib-0087] Rosenberger G , Liu Y , Rost HL , Ludwig C , Buil A , Bensimon A , Soste M , Spector TD , Dermitzakis ET , Collins BC , Malmstrom L , Aebersold R (2017b) Inference and quantification of peptidoforms in large sample cohorts by SWATH‐MS. Nat Biotechnol 35: 781–788 28604659 10.1038/nbt.3908 PMC5593115

[msb178126-bib-0088] Rost H , Malmstrom L , Aebersold R (2012) A computational tool to detect and avoid redundancy in selected reaction monitoring. Mol Cell Proteomics 11: 540–549 22535207 10.1074/mcp.M111.013045 PMC3412981

[msb178126-bib-0089] Rost HL , Rosenberger G , Navarro P , Gillet L , Miladinovic SM , Schubert OT , Wolski W , Collins BC , Malmstrom J , Malmstrom L , Aebersold R (2014) OpenSWATH enables automated, targeted analysis of data‐independent acquisition MS data. Nat Biotechnol 32: 219–223 24727770 10.1038/nbt.2841

[msb178126-bib-0090] Rost HL , Liu Y , D'Agostino G , Zanella M , Navarro P , Rosenberger G , Collins BC , Gillet L , Testa G , Malmstrom L , Aebersold R (2016a) TRIC: an automated alignment strategy for reproducible protein quantification in targeted proteomics. Nat Methods 13: 777–783 27479329 10.1038/nmeth.3954 PMC5008461

[msb178126-bib-0091] Rost HL , Sachsenberg T , Aiche S , Bielow C , Weisser H , Aicheler F , Andreotti S , Ehrlich HC , Gutenbrunner P , Kenar E , Liang X , Nahnsen S , Nilse L , Pfeuffer J , Rosenberger G , Rurik M , Schmitt U , Veit J , Walzer M , Wojnar D *et al* (2016b) OpenMS: a flexible open‐source software platform for mass spectrometry data analysis. Nat Methods 13: 741–748 27575624 10.1038/nmeth.3959

[msb178126-bib-0092] Roumeliotis TI , Williams SP , Gonçalves E , Alsinet C , Del Castillo Velasco‐Herrera M , Aben N , Ghavidel FZ , Michaut M , Schubert M , Price S , Wright JC , Yu L , Yang M , Dienstmann R , Guinney J , Beltrao P , Brazma A , Pardo M , Stegle O , Adams DJ *et al* (2017) Genomic determinants of protein abundance variation in colorectal cancer cells. Cell Rep 20: 2201–2214 28854368 10.1016/j.celrep.2017.08.010 PMC5583477

[msb178126-bib-0093] Rudnick PA , Clauser KR , Kilpatrick LE , Tchekhovskoi DV , Neta P , Blonder N , Billheimer DD , Blackman RK , Bunk DM , Cardasis HL , Ham AJ , Jaffe JD , Kinsinger CR , Mesri M , Neubert TA , Schilling B , Tabb DL , Tegeler TJ , Vega‐Montoto L , Variyath AM *et al* (2010) Performance metrics for liquid chromatography‐tandem mass spectrometry systems in proteomics analyses. Mol Cell Proteomics 9: 225–241 19837981 10.1074/mcp.M900223-MCP200 PMC2830836

[msb178126-bib-0094] Savitski MM , Mathieson T , Zinn N , Sweetman G , Doce C , Becher I , Pachl F , Kuster B , Bantscheff M (2013) Measuring and managing ratio compression for accurate iTRAQ/TMT quantification. J Proteome Res 12: 3586–3598 23768245 10.1021/pr400098r

[msb178126-bib-0095] Schmidlin T , Garrigues L , Lane CS , Mulder TC , van Doorn S , Post H , de Graaf EL , Lemeer S , Heck AJ , Altelaar AF (2016) Assessment of SRM, MRM and DIA for the targeted analysis of phosphorylation dynamics in non‐small cell lung cancer. Proteomics 16: 2193–2205 27219855 10.1002/pmic.201500453

[msb178126-bib-0096] Schubert OT , Mouritsen J , Ludwig C , Rost HL , Rosenberger G , Arthur PK , Claassen M , Campbell DS , Sun Z , Farrah T , Gengenbacher M , Maiolica A , Kaufmann SH , Moritz RL , Aebersold R (2013) The Mtb proteome library: a resource of assays to quantify the complete proteome of Mycobacterium tuberculosis. Cell Host Microbe 13: 602–612 23684311 10.1016/j.chom.2013.04.008 PMC3766585

[msb178126-bib-0097] Schubert OT , Gillet LC , Collins BC , Navarro P , Rosenberger G , Wolski WE , Lam H , Amodei D , Mallick P , MacLean B , Aebersold R (2015a) Building high‐quality assay libraries for targeted analysis of SWATH MS data. Nat Protoc 10: 426–441 25675208 10.1038/nprot.2015.015

[msb178126-bib-0098] Schubert OT , Ludwig C , Kogadeeva M , Zimmermann M , Rosenberger G , Gengenbacher M , Gillet LC , Collins BC , Rost HL , Kaufmann SH , Sauer U , Aebersold R (2015b) Absolute proteome composition and dynamics during dormancy and resuscitation of *Mycobacterium tuberculosis* . Cell Host Microbe 18: 96–108 26094805 10.1016/j.chom.2015.06.001

[msb178126-bib-0099] Schwanhausser B , Busse D , Li N , Dittmar G , Schuchhardt J , Wolf J , Chen W , Selbach M (2011) Global quantification of mammalian gene expression control. Nature 473: 337–342 21593866 10.1038/nature10098

[msb178126-bib-0100] Searle BC , Egertson JD , Bollinger JG , Stergachis AB , MacCoss MJ (2015) Using data independent acquisition (DIA) to model high‐responding peptides for targeted proteomics experiments. Mol Cell Proteomics 14: 2331–2340 26100116 10.1074/mcp.M115.051300 PMC4563719

[msb178126-bib-0101] Selevsek N , Chang CY , Gillet LC , Navarro P , Bernhardt OM , Reiter L , Cheng LY , Vitek O , Aebersold R (2015) Reproducible and consistent quantification of the *Saccharomyces cerevisiae* proteome by SWATH‐mass spectrometry. Mol Cell Proteomics 14: 739–749 25561506 10.1074/mcp.M113.035550 PMC4349991

[msb178126-bib-0102] Sharma V , Eckels J , Taylor GK , Shulman NJ , Stergachis AB , Joyner SA , Yan P , Whiteaker JR , Halusa GN , Schilling B , Gibson BW , Colangelo CM , Paulovich AG , Carr SA , Jaffe JD , MacCoss MJ , MacLean B (2014) Panorama: a targeted proteomics knowledge base. J Proteome Res 13: 4205–4210 25102069 10.1021/pr5006636 PMC4156235

[msb178126-bib-0103] Sidoli S , Lin S , Xiong L , Bhanu NV , Karch KR , Johansen E , Hunter C , Mollah S , Garcia BA (2015) Sequential window acquisition of all theoretical mass spectra (SWATH) analysis for characterization and quantification of histone post‐translational modifications. Mol Cell Proteomics 14: 2420–2428 25636311 10.1074/mcp.O114.046102 PMC4563725

[msb178126-bib-0104] Silva JC , Gorenstein MV , Li GZ , Vissers JP , Geromanos SJ (2006) Absolute quantification of proteins by LCMSE: a virtue of parallel MS acquisition. Mol Cell Proteomics 5: 144–156 16219938 10.1074/mcp.M500230-MCP200

[msb178126-bib-0105] Sonnett M , Yeung E , Wuhr M (2018) Accurate, sensitive, and precise multiplexed proteomics using the complement reporter ion cluster. Anal Chem 90: 5032–5039 29522331 10.1021/acs.analchem.7b04713 PMC6220677

[msb178126-bib-0106] Stergachis AB , MacLean B , Lee K , Stamatoyannopoulos JA , MacCoss MJ (2011) Rapid empirical discovery of optimal peptides for targeted proteomics. Nat Methods 8: 1041–1043 22056677 10.1038/nmeth.1770 PMC3227787

[msb178126-bib-0107] Sturm M , Bertsch A , Gropl C , Hildebrandt A , Hussong R , Lange E , Pfeifer N , Schulz‐Trieglaff O , Zerck A , Reinert K , Kohlbacher O (2008) OpenMS ‐ an open‐source software framework for mass spectrometry. BMC Bioinformatics 9: 163 18366760 10.1186/1471-2105-9-163 PMC2311306

[msb178126-bib-0108] Tan SLW , Chadha S , Liu Y , Gabasova E , Perera D , Ahmed K , Constantinou S , Renaudin X , Lee M , Aebersold R , Venkitaraman AR (2017) A class of environmental and endogenous toxins induces BRCA2 haploinsufficiency and genome instability. Cell 169: 1105–1118.e15 28575672 10.1016/j.cell.2017.05.010 PMC5457488

[msb178126-bib-0109] Teleman J , Rost HL , Rosenberger G , Schmitt U , Malmstrom L , Malmstrom J , Levander F (2015) DIANA–algorithmic improvements for analysis of data‐independent acquisition MS data. Bioinformatics 31: 555–562 25348213 10.1093/bioinformatics/btu686

[msb178126-bib-0110] Teleman J , Hauri S , Malmstrom J (2017) Improvements in mass spectrometry assay library generation for targeted proteomics. J Proteome Res 16: 2384–2392 28516777 10.1021/acs.jproteome.6b00928

[msb178126-bib-0111] Teo G , Kim S , Tsou CC , Collins B , Gingras AC , Nesvizhskii AI , Choi H (2015) mapDIA: preprocessing and statistical analysis of quantitative proteomics data from data independent acquisition mass spectrometry. J Proteomics 129: 108–120 26381204 10.1016/j.jprot.2015.09.013 PMC4630088

[msb178126-bib-0112] Thompson A , Schafer J , Kuhn K , Kienle S , Schwarz J , Schmidt G , Neumann T , Johnstone R , Mohammed AK , Hamon C (2003) Tandem mass tags: a novel quantification strategy for comparative analysis of complex protein mixtures by MS/MS. Anal Chem 75: 1895–1904 12713048 10.1021/ac0262560

[msb178126-bib-0113] Ting L , Rad R , Gygi SP , Haas W (2011) MS3 eliminates ratio distortion in isobaric multiplexed quantitative proteomics. Nat Methods 8: 937–940 21963607 10.1038/nmeth.1714 PMC3205343

[msb178126-bib-0114] Ting YS , Egertson JD , Payne SH , Kim S , MacLean B , Kall L , Aebersold R , Smith RD , Noble WS , MacCoss MJ (2015) Peptide‐centric proteome analysis: an alternative strategy for the analysis of tandem mass spectrometry data. Mol Cell Proteomics 14: 2301–2307 26217018 10.1074/mcp.O114.047035 PMC4563716

[msb178126-bib-0115] Ting YS , Egertson JD , Bollinger JG , Searle BC , Payne SH , Noble WS , MacCoss MJ (2017) PECAN: library‐free peptide detection for data‐independent acquisition tandem mass spectrometry data. Nat Methods 14: 903–908 28783153 10.1038/nmeth.4390 PMC5578911

[msb178126-bib-0116] Toprak UH , Gillet LC , Maiolica A , Navarro P , Leitner A , Aebersold R (2014) Conserved peptide fragmentation as a benchmarking tool for mass spectrometers and a discriminating feature for targeted proteomics. Mol Cell Proteomics 13: 2056–2071 24623587 10.1074/mcp.O113.036475 PMC4125737

[msb178126-bib-0117] Tsou CC , Avtonomov D , Larsen B , Tucholska M , Choi H , Gingras AC , Nesvizhskii AI (2015) DIA‐Umpire: comprehensive computational framework for data‐independent acquisition proteomics. Nat Methods 12: 258–264 25599550 10.1038/nmeth.3255 PMC4399776

[msb178126-bib-0118] Unwin RD , Pierce A , Watson RB , Sternberg DW , Whetton AD (2005) Quantitative proteomic analysis using isobaric protein tags enables rapid comparison of changes in transcript and protein levels in transformed cells. Mol Cell Proteomics 4: 924–935 15849271 10.1074/mcp.M400193-MCP200

[msb178126-bib-0119] Venable JD , Dong MQ , Wohlschlegel J , Dillin A , Yates JR (2004) Automated approach for quantitative analysis of complex peptide mixtures from tandem mass spectra. Nat Methods 1: 39–45 15782151 10.1038/nmeth705

[msb178126-bib-0120] Vowinckel J , Zelezniak A , Bruderer R , Mulleder M , Reiter L , Ralser M (2018) Cost‐effective generation of precise label‐free quantitative proteomes in high‐throughput by microLC and data‐independent acquisition. Sci Rep 8: 4346 29531254 10.1038/s41598-018-22610-4 PMC5847575

[msb178126-bib-0121] Wang X , Chambers MC , Vega‐Montoto LJ , Bunk DM , Stein SE , Tabb DL (2014) QC metrics from CPTAC raw LC‐MS/MS data interpreted through multivariate statistics. Anal Chem 86: 2497–2509 24494671 10.1021/ac4034455 PMC3982976

[msb178126-bib-0122] Wang J , Tucholska M , Knight JD , Lambert JP , Tate S , Larsen B , Gingras AC , Bandeira N (2015) MSPLIT‐DIA: sensitive peptide identification for data‐independent acquisition. Nat Methods 12: 1106–1108 26550773 10.1038/nmeth.3655 PMC4857761

[msb178126-bib-0123] Weisbrod CR , Eng JK , Hoopmann MR , Baker T , Bruce JE (2012) Accurate peptide fragment mass analysis: multiplexed peptide identification and quantification. J Proteome Res 11: 1621–1632 22288382 10.1021/pr2008175 PMC3319072

[msb178126-bib-0124] Whiteaker JR , Halusa GN , Hoofnagle AN , Sharma V , MacLean B , Yan P , Wrobel JA , Kennedy J , Mani DR , Zimmerman LJ , Meyer MR , Mesri M , Rodriguez H , Paulovich AG (2014) CPTAC assay portal: a repository of targeted proteomic assays. Nat Methods 11: 703–704 24972168 10.1038/nmeth.3002 PMC4113142

[msb178126-bib-0125] Wilhelm M , Schlegl J , Hahne H , Moghaddas Gholami A , Lieberenz M , Savitski MM , Ziegler E , Butzmann L , Gessulat S , Marx H , Mathieson T , Lemeer S , Schnatbaum K , Reimer U , Wenschuh H , Mollenhauer M , Slotta‐Huspenina J , Boese JH , Bantscheff M , Gerstmair A *et al* (2014) Mass‐spectrometry‐based draft of the human proteome. Nature 509: 582–587 24870543 10.1038/nature13319

[msb178126-bib-0126] Williams EG , Wu Y , Jha P , Dubuis S , Blattmann P , Argmann CA , Houten SM , Amariuta T , Wolski W , Zamboni N , Aebersold R , Auwerx J (2016) Systems proteomics of liver mitochondria function. Science 352: aad0189 27284200 10.1126/science.aad0189 PMC10859670

[msb178126-bib-0127] Wu JX , Song X , Pascovici D , Zaw T , Care N , Krisp C , Molloy MP (2016) SWATH mass spectrometry performance using extended peptide MS/MS assay libraries. Mol Cell Proteomics 15: 2501–2514 27161445 10.1074/mcp.M115.055558 PMC4937520

[msb178126-bib-0128] Zhang Y , Bilbao A , Bruderer T , Luban J , Strambio‐De‐Castillia C , Lisacek F , Hopfgartner G , Varesio E (2015) The use of variable Q1 isolation windows improves selectivity in LC‐SWATH‐MS acquisition. J Proteome Res 14: 4359–4371 26302369 10.1021/acs.jproteome.5b00543

[msb178126-bib-0129] Zi J , Zhang S , Zhou R , Zhou B , Xu S , Hou G , Tan F , Wen B , Wang Q , Lin L , Liu S (2014) Expansion of the ion library for mining SWATH‐MS data through fractionation proteomics. Anal Chem 86: 7242–7246 24969961 10.1021/ac501828a

[msb178126-bib-0130] Zolg DP , Wilhelm M , Schnatbaum K , Zerweck J , Knaute T , Delanghe B , Bailey DJ , Gessulat S , Ehrlich HC , Weininger M , Yu P , Schlegl J , Kramer K , Schmidt T , Kusebauch U , Deutsch EW , Aebersold R , Moritz RL , Wenschuh H , Moehring T *et al* (2017) Building ProteomeTools based on a complete synthetic human proteome. Nat Methods 14: 259–262 28135259 10.1038/nmeth.4153 PMC5868332

[msb178126-bib-0131] Zybailov B , Mosley AL , Sardiu ME , Coleman MK , Florens L , Washburn MP (2006) Statistical analysis of membrane proteome expression changes in *Saccharomyces cerevisiae* . J Proteome Res 5: 2339–2347 16944946 10.1021/pr060161n

