# Supplementary material for: Data‐independent acquisition‐based SWATH‐MS for quantitative proteomics: a tutorial
Source: Mol Syst Biol. 2018 Aug 13;14(8):e8126. doi: 10.15252/msb.20178126 (PMC6088389; doi:10.15252/msb.20178126)
Supplement: Supplementary file 1 — Appendix [file MSB-14-e8126-s001.pdf]

Appendix to

Data independent acquisition-based SWATH-MS for quantitative proteomics: a tutorial

Christina Ludwig [1#*], Ludovic Gillet [2#], George Rosenberger [2,3], Sabine Amon [2], Ben Collins [2], Ruedi Aebersold [2,4]

[#] authors contributed equally

[1] Bavarian Center for Biomolecular Mass Spectrometry (BayBioMS), Technical University of Munich (TUM), Freising, Germany

[2] Department of Biology, Institute of Molecular Systems Biology, ETH Zurich, Zurich, Switzerland

[3] Department of Systems Biology, Columbia University, New York, NY, USA

[4] Faculty of Science, University of Zurich, Zurich, Switzerland

* corresponding author

History and terminology of DIA and SWATH-MS

The fundamental idea of data-independent acquisition (DIA) is to record fragment ion mass spectra (MS2 spectra), regardless whether a peptide precursor ion is detected or not. This concept was published for the first time in a proof-of-principle experiment by Masselon *et al.* in the year 2000 (Masselon et al, 2000). Shortly after, several groups further developed this idea and demonstrated parallel fragmentation of all peptides (Bateman et al, 2002; Purvine et al, 2003) as well as the usage of sequentially windowed precursor isolation methods (Venable et al, 2004). Since then, different varieties of DIA have been explored and various acquisition schemes and analysis workflows have been implemented (Carvalho et al, 2010; Egertson et al, 2013; Geiger et al, 2010; Gillet et al, 2012; Panchaud et al, 2011; Panchaud et al, 2009; Plumb et al, 2006; Purvine et al, 2003; Silva et al, 2005; Venable et al, 2004; Weisbrod et al, 2012) (Figure S1). The methods differ in the type of mass spectrometer used, in the specific data acquisition parameters and in the data analysis strategies employed. For recent reviews that compare different DIA methods and discuss specific advantages and disadvantages see (Chapman et al, 2014; Law & Lim, 2013; Meyer & Schilling, 2017; Sajic et al, 2015).
The terminology “DIA” was originally coined by Venable *et al.* in 2004 (Venable et al, 2004), to contrast with data dependent acquisition (DDA) where peptide ions are specifically selected for fragmentation based on their detection in precursor ion scans. Initially, data generated by DIA was analysed using similar database search tools as for DDA data, either by searching the acquired mixed MS2 spectra directly (Panchaud et al, 2009; Venable et al,

2004) or by searching pseudo MS2 spectra that were reconstituted based on co-elution of precursor ions and their potential fragment ions (Bern et al, 2010; Geromanos et al, 2009; Li et al, 2009). A new era in the field started 2012, when Gillet *et al.* proposed a novel query strategy based on prior knowledge as an alternative DIA data analysis workflow (Gillet et al, 2012). In a method analogous to targeted proteomics by selected/multiple reaction monitoring (S/MRM), Gillet *et al.* used a consecutive acquisition scheme that supports the repeated measurement of fragment ions of the same peptide over the chromatographic elution time and exploited prior information from spectral libraries, in which mass spectrometric and chromatographic parameters from previously observed peptides are used to query the data. The authors termed their method SWATH-MS in analogy to the field of geophysics, where “swaths” refers to image strips of the Earth’s surface acquired by a satellite or aircraft and where the acquired “swaths” collectively can be reconstructed *in silico* into a complete map of the earth. In SWATH-MS a “swath” refers to a strip of the peptide mass range selected for fragmentation by the mass spectrometer corresponding to a specific width of the precursor isolation window.

Due to important advancements in data analysis and technical improvements in acquisition speed, mass accuracy and resolution, scientific interest and research activity in the field of DIA have increased dramatically over the last few years (Doerr, 2015). Numerous variants of DIA methods have been developed (Figure S1) and several automated DIA data analysis tools have been implemented (Bilbao et al, 2015). This allows proteomic laboratories to perform routine high-quality DIA experiments and to apply DIA methods successfully in various research areas (Aebersold et al, 2016).

In the wealth of DIA acquisition schemes and data analysis strategies, several different names have been coined and are now in use. The naming situation is further complicated by trademarking, whereby certain names are associated with specific mass spectrometer vendors. Throughout the tutorial we will use the generic term “DIA” when referring to the breadth of all data-independent acquisition strategies, while we use the term “SWATH-MS” when referring to the specific acquisition and data analysis method described by Gillet *et al.*(Gillet et al, 2012) and which is the focus of this tutorial.

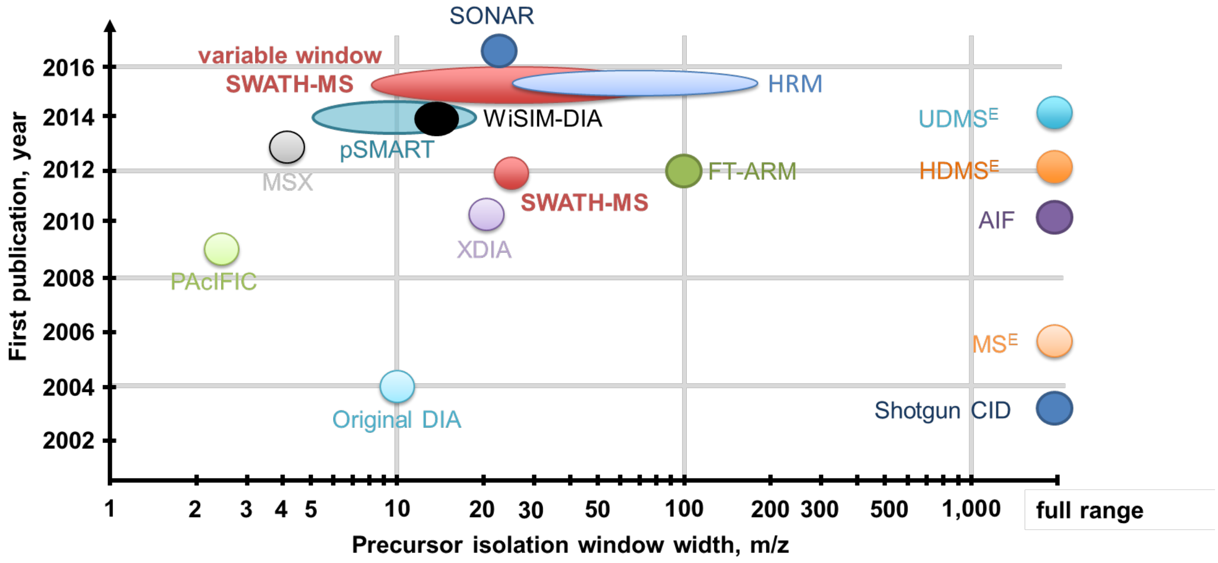

Figure S1: Representation of the historical development of DIA methods with their corresponding precursor isolation window widths. Since the introduction of DIA in the early 2000s different implementations of DIA have emerged. They differ in the instrument type used, the data analysis strategy and specific acquisition parameters such as the precursor isolation window widths. Due to technical improvements in instrument acquisition speed and resolution, in addition to novel data analysis approaches, research activity in the field has dramatically increased over the last few years. Several novel DIA approaches with "medium-sized" precursor isolation window widths (in the range between 4 and 100 m/z) have been published recently. References: Shotgun-CID (Purvine et al, 2003), Original DIA (Venable et al, 2004) , MS^E (Silva et al, 2006), PAcIFIC (Panchaud et al, 2009), XDIA (Carvalho et al, 2010), AIF (Geiger et al, 2010), SWATH-MS (Gillet et al, 2012), FT-ARM (Weisbrod et al, 2012), $HDMS^E$ (Geromanos et al, 2012), MSX (Egertson et al, 2013), pSMART (Prakash et al, 2014), WiSIM-DIA (Martin et al, 2016), $UDMS^E$ (Distler et al, 2014), HRM (Bruderer et al, 2015), variable window SWATH-MS (Zhang et al, 2015), SONAR (Moseley et al, 2018).

The basic principle of SWATH-MS

SWATH-MS measurements can be performed on fast scanning (usually at least 10 Hz), high-resolution (usually at least 15000 at full width half maximum on MS2 level) and accurate mass (usually at least 50 ppm) hybrid mass spectrometers, typically employing a quadrupole (Q) as first mass analyser and a time-of-flight (TOF) or Orbitrap as second mass analyser (Figure 1A). Technical improvements in quadrupole design have been realized in recent years that enable efficient and even transmission of precursors across the entire width of a SWATH precursor isolation window resulting in a "squared" precursor isolation window (Figure 4B). This important feature assures that the intensity of an acquired signal and thus the quantity determined for the specific analyte is independent of the position of the precursor in the isolation window.
During SWATH-MS data acquisition, series of MS2 scans are recorded repeatedly during the entire chromatographic gradient. Optionally, one or several MS1 scans per cycle can be included (Figure 1A). Importantly, all MS2 scans are independent from the content of the optional MS1 scans. In every MS2 scan a wide precursor window is isolated. This precursor

isolation window is adjusted stepwise in defined increments, until a predefined total precursor ion mass range is covered (Figure 1A). To ensure a sufficiently fast recording of MS1 and MS2 scans for chromatogram extraction and peptide quantification, an optimal balance between the number of MS2 scans, the accumulation time per scan, the precursor isolation window width, and the peptide mass range must be found. For example, the SWATH-MS acquisition scheme originally published by Gillet *et al.* used 32 precursor isolation windows of 25 m/z each to cover a range from 400 to 1200 m/z with an accumulation time per scan of 100 ms (Figure 1B).

One important consequence arising from such wide precursor isolation windows is that co-eluting peptide precursors falling into the same precursor isolation window will be co-fragmented (Figure 1C and 1D). Depending on sample complexity this can lead to 10s or even 100s of co-fragmented peptides (Rost et al, 2012), which results in highly mixed and convoluted MS2 spectra. Such multiplexed MS2 spectra can no longer be successfully analysed using the established database search tools developed for the analysis of MS2 spectra generated by DDA. Instead SWATH-MS data require advanced data processing strategies. Of course, convoluted fragment ion spectra from co-fragmentation of more than one precursor are also observed in DDA-based and targeted proteomics where isolation window widths range from 0.5 to 3 m/z, but to a significantly smaller extent (Michalski et al, 2011; Wang et al, 2014). One out of several possible DIA analysis strategies developed for SWATH-MS data is “peptide-centric scoring” (Ting et al, 2015). In this strategy the acquired dataset is queried for the presence of a specific peptide using a set of peptide query parameters that relate to such peptides. This is most typically achieved by using previously generated knowledge in form of spectral libraries. This strategy, also referred to as “targeted data extraction” was described in the initial SWATH-MS publication (Gillet et al, 2012) and has remained the primary approach for DIA data analysis in applied projects to date.

In summary, in SWATH-MS all ionized peptides that fall within the isolated precursor mass range are fragmented and recorded in a systematic and unbiased fashion. Qualitative and quantitative information in SWATH-MS data needs to be extracted from highly multiplexed MS2 spectra, which requires advanced processing strategies, such as peptide-centric scoring. Altogether, SWATH-MS is a good compromise between the proteome-coverage capabilities of DDA-based proteomics and the quantitative consistency of targeted proteomics.

Advantages of SWATH-MS

The principles of SWATH-MS outlined above result in specific performance benefits and limitations, which are summarized and compared in Table 1. The favourable performance characteristics of SWATH-MS and other DIA-based methods have contributed to its recent popularization (Doerr, 2015) and will be discussed in the following paragraphs.

Ease of data acquisition

An important advantage of SWATH-MS is the simplicity of data acquisition. A SWATH-MS measurement requires definition of the precursor isolation scheme, which includes parameters such as precursor isolation window widths, m/z range to cover, and accumulation time for the MS2 scans. Once this acquisition scheme has been optimized for a certain sample type on a given LC-MS/MS system, all samples of that particular type can be analysed with the same method. The acquisition scheme has also proven quite robust across sample types. This ease in data acquisition is similar to DDA acquisition, and an advantage over targeted data acquisition where peptide precursor ions (PRM) or peptide-fragment ion pairs (SRM) need to be defined upfront for every peptide of interest and where data acquisition often needs to be scheduled according to retention time (Picotti & Aebersold, 2012).

Breadth of peptide detection and multiplexing

A second advantage of SWATH-MS is the high multiplexing rate achievable for peptide identification and quantification. Similar to DDA-based methods, but in contrast to targeted proteomics, it is possible to query and detect 10,000s of peptides in a single SWATH-MS injection. This has been demonstrated for example for human cell lines such as HEK293, where routinely 30,000-40,000 peptides were identified from which 4000-5000 proteins were inferred at a 1% protein FDR cut off in a 2-hour run (Collins et al, 2017). Therefore, SWATH-MS provides a dramatic increase in the number of peptides that are quantified in a single run when compared with targeted proteomics by SRM or PRM. The higher number of quantified peptides leads to a higher number of inferred proteins and in increased coverage of their primary amino acid sequence. This can improve protein inference, can lead to more precise and accurate protein quantification and may allow a more extensive mapping of posttranslational modifications or protein variants. Furthermore, a high multiplexing rate is beneficial for global data normalisation. With SRM and PRM, global normalization between samples is often difficult, because the underlying assumption that the major fraction of the detected proteome does not change its concentration between samples is often not true (especially if the target peptides were selected based on a prior hypothesis of being involved in the studied biological process). SWATH-MS, just like DDA, allows parallel monitoring of a potentially unbiased set of peptides with regards to regulation, which is necessary to perform global normalisation.

Reproducibility and consistency

When proteomic studies take on a scale of hundreds of samples, reproducibility of peptide detection and quantification becomes a major issue. In DDA-based proteomics consistency in peptide sequencing is difficult due to the heuristic precursor selection carried out by the mass spectrometer in real-time. Therefore, when a peptide is not identified in a particular DDA measurement, it not justified to conclude that this peptide is not present at a detectable level in the sample (“true negative peptide quantity”). Instead it might be that this peptide was not selected for fragmentation due to the presence of many co-eluting peptides (“false negative peptide quantity”) (Michalski et al, 2011). An important

improvement, which helped to decrease the number of such false negatives, was the development of analysis tools that allow to transfer peptide identifications between samples and thereby to improve the completeness of the quantitative data matrix (Cox et al, 2014; Mueller et al, 2007; Prakash et al, 2006).

In contrast, in SRM, PRM and SWATH-MS, MS2 data acquisition is performed in a systematic fashion and a peptide centric analysis strategy is used. In combination, these strategies make it possible to conclude with a higher confidence that a targeted peptide is correctly "not detected" and therefore indeed not present in the sample at a concentration above the lower limit of detection. Nonetheless, when automated data analysis tools are used to query thousands of proteins, or tens of thousands of peptides, also in SWATH-MS data "false negative peptide quantities" will occur to some extent. However, just as for DDA, also for SWATH-MS software tools have been established (Rost et al, 2016) that allow to align SWATH-MS runs and to transfer identifications between runs. Due to the increased sensitivity of fragment ion detection compared to precursor ion detection, as well as due to the higher information content of MS2 data (co-elution of many fragment ion chromatograms compared to just one precursor ion chromatogram), this transfer of identifications can be more robust and sensitive for SWATH-MS than for DDA.

Finally, for SRM (Abbatiello et al, 2015; Addona et al, 2009) as well as SWATH-MS (Collins et al, 2017) excellent intra- and inter-laboratory reproducibility have been reported, with coefficients of variance (CV) below 20% CV, even in large-scale studies including several international laboratories. Consequently, such high levels of reproducibility and consistency lead to high-quality quantitative data matrices that are suited to address a wide variety of biological questions (Rost et al, 2015).

Retrospective querying

SWATH-MS data is comprehensive in the chromatographic and mass dimensions in both the MS1 precursor and MS2 fragment ion spaces. This is in contrast to DDA data, where precursor ions are continuously recorded on MS1 level, but not so the measurements of fragment ions and the MS2 data is hence inherently incomplete. SWATH-MS data are well suited to be re-analysed at a later time point, when new proteins, peptides or PTMs, that were not part of the first biological hypothesis or for which peptide query parameters were initially not available, should be included (Rosenberger et al, 2017). This is unlike SRM or PRM analyses, where for novel target proteins or peptides new measurements are required.

Analysis of modified peptides

An additional promise of SWATH-MS is its usefulness to identify and quantify modified peptides, to localise the amino acid position of a modification within a peptide sequence and to generally search for previously unexpected analytes. These important tasks are supported in SWATH-MS by the high-resolution and accurate mass MS2 spectra that are recorded recursively over the whole elution profile of a peptide. Therefore, maximally informative XICs can be extracted from the data at the precursor (MS1) and fragment ion (MS2) level without concerns of stochastic sampling or dynamic exclusion that might hinder

identification of isobaric species, as is the case in DDA. Furthermore, for the purpose of PTM-site localisation, fragment ions that are deterministic for the position of the modified amino acid can be extracted and used to discriminate between possible sites of modification, because full MS2 spectra are available (Keller et al, 2016; Meyer et al, 2017; Rosenberger et al, 2017). Finally, SWATH-MS in combination with open modification search tools offers unique possibilities to discover unanticipated modifications through the peptide-centric query strategy, which does not suffer from the combinatorial explosion of the search space (Keller et al, 2016; Wang et al, 2015). However, intrinsic challenges when analyzing modified peptides apply to SWATH-MS in the same way as to any other bottom-up proteomic approach: the possibly low modification stoichiometry and the therefore required high dynamic quantification range, as modified peptides tend to be much lower concentrated than other peptides in the sample.

In summary, the above mentioned performance characteristics make SWATH-MS ideally suited for projects that require accurate quantitative information for a large fraction of peptides in a given sample and that include large sample cohorts to be analysed in a maximally reproducible and consistent way. Typical projects that require these properties include systems biology studies (Bensimon et al, 2012), genetic association studies (Liu et al, 2015; Okada et al, 2016; Williams et al, 2016) clinical screens (Liu et al, 2014; Sajic et al, 2015), drug/perturbation screens (Litichevskiy et al, 2017) or exploratory basic research (Collins et al, 2013; Lambert et al, 2013; Parker et al, 2015; Schubert et al, 2015b; Selevsek et al, 2015). SWATH-MS is also well suited for fast analyses, where proteome coverage of ~50% of the MS-detectable proteome in complex mammalian samples can be achieved in a single shot analyses (Bruderer et al, 2017)

Limitations and challenges of SWATH-MS

Prior knowledge and the availability of a spectral library

In the most routinely and successfully applied SWATH-MS workflows, peptide-centric scoring requires prior knowledge about the chromatographic and mass spectrometric behaviour of the queried peptides in the form of a high-quality spectral library (Schubert et al, 2015a). Usually spectral libraries are generated from discovery proteomics data obtained by DDA, which means that only peptides that were previously detected are contained in the spectral library. In recent years, alternative data analysis approaches for SWATH-MS data that do not require spectral libraries have been developed, examples are DIA-Umpire (Tsou et al, 2015), FT-ARM (Weisbrod et al, 2012), and PECAN (Ting et al, 2017).

Ease of data analysis

Currently the main challenge in SWATH-MS experiments is the peptide-centric data analysis part. The highly multiplexed MS2 spectra originating from 10s to 100s of co-fragmented peptide precursor ions require a sophisticated analysis pipeline to detect and quantify peptides and assign a measure of statistical confidence. For DDA-based proteomics a wealth

of analysis pipelines have been developed over the past ~20 years (Cox & Mann, 2008; Deutsch et al, 2015; Keller et al, 2005; Reinert & Kohlbacher, 2010). Also for targeted proteomics mature software tools have been readily available for several years (Colangelo et al, 2013; Malmstrom et al, 2012; Teleman et al, 2012). In comparison, automated peptide-centric SWATH-MS analysis is at an earlier stage of development, but several analysis tools are readily available, some of them freely as open source software (for a recent review see (Bilbao et al, 2015)). A comparison of five widely used tools shows that they largely agree in their detection and quantification results (Navarro et al, 2016).

Selectivity, sensitivity and dynamic quantification range

A major challenge of SWATH-MS, as of any other mass spectrometric approach, is the limited dynamic quantification range afforded by the instruments. Several groups have reported that in complex proteomic samples quantification by fragment ion signals (MS2) is more sensitive than by the corresponding precursor signals (MS1) due to a better signal-to-noise ratio, selectivity, and intra-scan dynamic range (Egertson et al, 2013; Gillet et al, 2012; Venable et al, 2004). In complex samples there is a greater likelihood that an MS1 signal for a given peptide will be interfered by the signal of another analyte with the same or very similar m/z value. Signals at the MS2 level, on the other hand, are less prone to interferences due to the additional filtering step of precursor isolation and the fact that several fragment ion signals can be combined for quantification. In addition to selectivity considerations, in trapping instruments, a gain in sensitivity can be expected because the signal for a given peptide in MS1 scans may be limited by the automatic gain control effects driven by other more abundant peptides from across the mass range, whereas in SWATH-MS this effect is significantly reduced and limited only to peptides co-isolated in the same SWATH precursor isolation window. In its current implementation on a Q-TOF instrument, SWATH-MS covers a peptide concentration range of 4.0 to 4.5 orders of magnitude in a single injection of a complex proteomic sample, such as a whole-cell lysate of a human cell line (Collins et al, 2017). Reported lower limits of quantification in this complex sample are in the mid-attomole to low femtomole range (on-column). Such a quantification range is 3- to 10-fold less sensitive than in state-of-the-art SRM or PRM measurements (Gillet et al, 2012; Liu et al, 2013; Schmidlin et al, 2016) .

In conclusion, a current drawback of SWATH-MS compared to SRM or PRM is that peptide quantification with SWATH-MS is still less sensitive. Hence, for projects that involve quantification of especially low abundant peptides with maximal accuracy, targeted data acquisition still remains the better option, especially if only few peptides are of interest. Further, SWATH-MS measurements require some upfront effort on spectral library and peptide query parameter generation and optimization.

References Appendix:

Abbatiello SE, Schilling B, Mani DR, Zimmerman LJ, Hall SC, MacLean B, Albertolle M, Allen S, Burgess M, Cusack MP, Gosh M, Hedrick V, Held JM, Inerowicz HD, Jackson A, Keshishian H,

Kinsinger CR, Lyssand J, Makowski L, Mesri M et al (2015) Large-Scale Interlaboratory Study to Develop, Analytically Validate and Apply Highly Multiplexed, Quantitative Peptide Assays to Measure Cancer-Relevant Proteins in Plasma. *Molecular & cellular proteomics : MCP* **14:** 2357-2374

Addona TA, Abbatiello SE, Schilling B, Skates SJ, Mani DR, Bunk DM, Spiegelman CH, Zimmerman LJ, Ham AJ, Keshishian H, Hall SC, Allen S, Blackman RK, Borchers CH, Buck C, Cardasis HL, Cusack MP, Dodder NG, Gibson BW, Held JM et al (2009) Multi-site assessment of the precision and reproducibility of multiple reaction monitoring-based measurements of proteins in plasma. *Nature biotechnology* **27:** 633-641

Aebersold R, Bensimon A, Collins BC, Ludwig C, Sabido E (2016) Applications and Developments in Targeted Proteomics: From SRM to DIA/SWATH. *Proteomics* **16:** 2065-2067

Bateman RH, Carruthers R, Hoyes JB, Jones C, Langridge JI, Millar A, Vissers JP (2002) A novel precursor ion discovery method on a hybrid quadrupole orthogonal acceleration time-of-flight (Q-TOF) mass spectrometer for studying protein phosphorylation. *Journal of the American Society for Mass Spectrometry* **13:** 792-803

Bensimon A, Heck AJ, Aebersold R (2012) Mass spectrometry-based proteomics and network biology. *Annual review of biochemistry* **81:** 379-405

Bern M, Finney G, Hoopmann MR, Merrihew G, Toth MJ, MacCoss MJ (2010) Deconvolution of mixture spectra from ion-trap data-independent-acquisition tandem mass spectrometry. *Analytical chemistry* **82:** 833-841

Bilbao A, Varesio E, Luban J, Strambio-De-Castillia C, Hopfgartner G, Muller M, Lisacek F (2015) Processing strategies and software solutions for data-independent acquisition in mass spectrometry. *Proteomics* **15:** 964-980

Bruderer R, Bernhardt OM, Gandhi T, Miladinovic SM, Cheng LY, Messner S, Ehrenberger T, Zanotelli V, Butscheid Y, Escher C, Vitek O, Rinner O, Reiter L (2015) Extending the limits of quantitative proteome profiling with data-independent acquisition and application to acetaminophen-treated three-dimensional liver microtissues. *Molecular & cellular proteomics : MCP* **14:** 1400-1410

Bruderer R, Bernhardt OM, Gandhi T, Xuan Y, Sondermann J, Schmidt M, Gomez-Varela D, Reiter L (2017) Optimization of Experimental Parameters in Data-Independent Mass Spectrometry Significantly Increases Depth and Reproducibility of Results. *Molecular & cellular proteomics : MCP*

Carvalho PC, Han X, Xu T, Cociorva D, Carvalho Mda G, Barbosa VC, Yates JR, 3rd (2010) XDIA: improving on the label-free data-independent analysis. *Bioinformatics (Oxford, England)* **26:** 847-848

Chapman JD, Goodlett DR, Masselon CD (2014) Multiplexed and data-independent tandem mass spectrometry for global proteome profiling. *Mass spectrometry reviews* **33:** 452-470

Colangelo CM, Chung L, Bruce C, Cheung KH (2013) Review of software tools for design and analysis of large scale MRM proteomic datasets. *Methods (San Diego, Calif)* **61:** 287-298

Collins BC, Gillet LC, Rosenberger G, Rost HL, Vichalkovski A, Gstaiger M, Aebersold R (2013) Quantifying protein interaction dynamics by SWATH mass spectrometry: application to the 14-3-3 system. *Nature methods* **10:** 1246-1253

Collins BC, Hunter CL, Liu Y, Schilling B, Rosenberger G, Bader SL, Chan DW, Gibson BW, Gingras AC, Held JM, Hirayama-Kurogi M, Hou G, Krisp C, Larsen B, Lin L, Liu S, Molloy MP, Moritz RL, Ohtsuki S, Schlapbach R et al (2017) Multi-laboratory assessment of reproducibility, qualitative and quantitative performance of SWATH-mass spectrometry. *Nature communications* **8:** 291

Cox J, Hein MY, Luber CA, Paron I, Nagaraj N, Mann M (2014) Accurate proteome-wide label-free quantification by delayed normalization and maximal peptide ratio extraction, termed MaxLFQ. *Molecular & cellular proteomics : MCP* **13:** 2513-2526

Cox J, Mann M (2008) MaxQuant enables high peptide identification rates, individualized p.p.b.-range mass accuracies and proteome-wide protein quantification. *Nature biotechnology* **26:** 1367-1372

Deutsch EW, Mendoza L, Shteynberg D, Slagel J, Sun Z, Moritz RL (2015) Trans-Proteomic Pipeline, a standardized data processing pipeline for large-scale reproducible proteomics informatics. *Proteomics Clinical applications* **9:** 745-754

Distler U, Kuharev J, Navarro P, Levin Y, Schild H, Tenzer S (2014) Drift time-specific collision energies enable deep-coverage data-independent acquisition proteomics. *Nature methods* **11:** 167-170

Doerr A (2015) DIA mass spectrometry. *Nat Meth* **12:** 35-35

Egertson JD, Kuehn A, Merrihew GE, Bateman NW, MacLean BX, Ting YS, Canterbury JD, Marsh DM, Kellmann M, Zabrouskov V, Wu CC, MacCoss MJ (2013) Multiplexed MS/MS for improved data-independent acquisition. *Nature methods* **10:** 744-746

Geiger T, Cox J, Mann M (2010) Proteomics on an Orbitrap benchtop mass spectrometer using all-ion fragmentation. *Molecular & cellular proteomics : MCP* **9:** 2252-2261

Geromanos SJ, Hughes C, Ciavarini S, Vissers JP, Langridge JI (2012) Using ion purity scores for enhancing quantitative accuracy and precision in complex proteomics samples. *Analytical and bioanalytical chemistry* **404:** 1127-1139

Geromanos SJ, Vissers JP, Silva JC, Dorschel CA, Li GZ, Gorenstein MV, Bateman RH, Langridge JI (2009) The detection, correlation, and comparison of peptide precursor and product ions from data independent LC-MS with data dependant LC-MS/MS. *Proteomics* **9:** 1683-1695

Gillet LC, Navarro P, Tate S, Rost H, Selevsek N, Reiter L, Bonner R, Aebersold R (2012) Targeted data extraction of the MS/MS spectra generated by data-independent acquisition: a new concept for consistent and accurate proteome analysis. *Molecular & cellular proteomics : MCP* **11:** O111.016717

Keller A, Bader SL, Kusebauch U, Shteynberg D, Hood L, Moritz RL (2016) Opening a SWATH Window on Posttranslational Modifications: Automated Pursuit of Modified Peptides. *Molecular & cellular proteomics : MCP* **15:** 1151-1163

Keller A, Eng J, Zhang N, Li XJ, Aebersold R (2005) A uniform proteomics MS/MS analysis platform utilizing open XML file formats. *Molecular systems biology* **1:** 2005.0017

Lambert JP, Ivosev G, Couzens AL, Larsen B, Taipale M, Lin ZY, Zhong Q, Lindquist S, Vidal M, Aebersold R, Pawson T, Bonner R, Tate S, Gingras AC (2013) Mapping differential interactomes by affinity purification coupled with data-independent mass spectrometry acquisition. *Nature methods* **10:** 1239-1245

Law KP, Lim YP (2013) Recent advances in mass spectrometry: data independent analysis and hyper reaction monitoring. *Expert review of proteomics* **10:** 551-566

Li GZ, Vissers JP, Silva JC, Golick D, Gorenstein MV, Geromanos SJ (2009) Database searching and accounting of multiplexed precursor and product ion spectra from the data independent analysis of simple and complex peptide mixtures. *Proteomics* **9:** 1696-1719

Litichevskiy L, Peckner R, Abelin JG, Asiedu JK, Creech AL, Davis JF, Davison D, Dunning CM, Egertson JD, Egri S, Gould J, Ko T, Johnson SA, Lahr DL, Lam D, Liu Z, Lyons NJ, Lu X, MacLean BX, Mungenast AE et al (2017) A Library of Phosphoproteomic and Chromatin Signatures for Characterizing Cellular Responses to Drug Perturbations. *bioRxiv*

Liu Y, Buil A, Collins BC, Gillet LC, Blum LC, Cheng LY, Vitek O, Mouritsen J, Lachance G, Spector TD, Dermitzakis ET, Aebersold R (2015) Quantitative variability of 342 plasma proteins in a human twin population. *Molecular systems biology* **11:** 786

Liu Y, Chen J, Sethi A, Li QK, Chen L, Collins B, Gillet LC, Wollscheid B, Zhang H, Aebersold R (2014) Glycoproteomic analysis of prostate cancer tissues by SWATH mass spectrometry discovers N-acylethanolamine acid amidase and protein tyrosine kinase 7 as signatures for tumor aggressiveness. *Molecular & cellular proteomics : MCP* **13:** 1753-1768

Liu Y, Huttenhain R, Surinova S, Gillet LC, Mouritsen J, Brunner R, Navarro P, Aebersold R (2013) Quantitative measurements of N-linked glycoproteins in human plasma by SWATH-MS. *Proteomics* **13:** 1247-1256

Malmstrom L, Malmstrom J, Selevsek N, Rosenberger G, Aebersold R (2012) Automated workflow for large-scale selected reaction monitoring experiments. *Journal of proteome research* **11:** 1644-1653

Martin LB, Sherwood RW, Nicklay JJ, Yang Y, Muratore-Schroeder TL, Anderson ET, Thannhauser TW, Rose JK, Zhang S (2016) Application of wide selected-ion monitoring data-

independent acquisition to identify tomato fruit proteins regulated by the CUTIN DEFICIENT2 transcription factor. *Proteomics* **16:** 2081-2094

Masselon C, Anderson GA, Harkewicz R, Bruce JE, Pasa-Tolic L, Smith RD (2000) Accurate mass multiplexed tandem mass spectrometry for high-throughput polypeptide identification from mixtures. *Analytical chemistry* **72:** 1918-1924

Meyer JG, Mukkamalla S, Steen H, Nesvizhskii AI, Gibson BW, Schilling B (2017) PIQED: automated identification and quantification of protein modifications from DIA-MS data. *Nature methods* **14:** 646-647

Meyer JG, Schilling B (2017) Clinical applications of quantitative proteomics using targeted and untargeted data-independent acquisition techniques. *Expert review of proteomics* **14:** 419-429

Michalski A, Cox J, Mann M (2011) More than 100,000 detectable peptide species elute in single shotgun proteomics runs but the majority is inaccessible to data-dependent LC-MS/MS. *Journal of proteome research* **10:** 1785-1793

Moseley MA, Hughes CJ, Juvvadi PR, Soderblom EJ, Lennon S, Perkins SR, Thompson JW, Steinbach WJ, Geromanos SJ, Wildgoose J, Langridge JI, Richardson K, Vissers JPC (2018) Scanning Quadrupole Data-Independent Acquisition, Part A: Qualitative and Quantitative Characterization. *Journal of proteome research* **17:** 770-779

Mueller LN, Rinner O, Schmidt A, Letarte S, Bodenmiller B, Brusniak MY, Vitek O, Aebersold R, Muller M (2007) SuperHirn - a novel tool for high resolution LC-MS-based peptide/protein profiling. *Proteomics* **7:** 3470-3480

Navarro P, Kuharev J, Gillet LC, Bernhardt OM, MacLean B, Rost HL, Tate SA, Tsou CC, Reiter L, Distler U, Rosenberger G, Perez-Riverol Y, Nesvizhskii AI, Aebersold R, Tenzer S (2016) A multicenter study benchmarks software tools for label-free proteome quantification. *Nature biotechnology*

Okada H, Ebhardt HA, Vonesch SC, Aebersold R, Hafen E (2016) Proteome-wide association studies identify biochemical modules associated with a wing-size phenotype in Drosophila melanogaster. *Nature communications* **7:** 12649

Panchaud A, Jung S, Shaffer SA, Aitchison JD, Goodlett DR (2011) Faster, quantitative, and accurate precursor acquisition independent from ion count. *Analytical chemistry* **83:** 2250-2257

Panchaud A, Scherl A, Shaffer SA, von Haller PD, Kulasekara HD, Miller SI, Goodlett DR (2009) Precursor acquisition independent from ion count: how to dive deeper into the proteomics ocean. *Analytical chemistry* **81:** 6481-6488

Parker BL, Yang G, Humphrey SJ, Chaudhuri R, Ma X, Peterman S, James DE (2015) Targeted phosphoproteomics of insulin signaling using data-independent acquisition mass spectrometry. *Science signaling* **8:** rs6

Picotti P, Aebersold R (2012) Selected reaction monitoring-based proteomics: workflows, potential, pitfalls and future directions. *Nature methods* **9:** 555-566

Plumb RS, Johnson KA, Rainville P, Smith BW, Wilson ID, Castro-Perez JM, Nicholson JK (2006) UPLC/MS(E); a new approach for generating molecular fragment information for biomarker structure elucidation. *Rapid communications in mass spectrometry : RCM* **20:** 1989-1994

Prakash A, Mallick P, Whiteaker J, Zhang H, Paulovich A, Flory M, Lee H, Aebersold R, Schwikowski B (2006) Signal maps for mass spectrometry-based comparative proteomics. *Molecular & cellular proteomics : MCP* **5:** 423-432

Prakash A, Peterman S, Ahmad S, Sarracino D, Frewen B, Vogelsang M, Byram G, Krastins B, Vadali G, Lopez M (2014) Hybrid data acquisition and processing strategies with increased throughput and selectivity: pSMART analysis for global qualitative and quantitative analysis. *Journal of proteome research* **13:** 5415-5430

Purvine S, Eppel JT, Yi EC, Goodlett DR (2003) Shotgun collision-induced dissociation of peptides using a time of flight mass analyzer. *Proteomics* **3:** 847-850

Reinert K, Kohlbacher O (2010) OpenMS and TOPP: open source software for LC-MS data analysis. *Methods in molecular biology (Clifton, NJ)* **604:** 201-211

Rosenberger G, Liu Y, Rost HL, Ludwig C, Buil A, Bensimon A, Soste M, Spector TD, Dermitzakis ET, Collins BC, Malmstrom L, Aebersold R (2017) Inference and quantification of peptidoforms in large sample cohorts by SWATH-MS. *Nature biotechnology* **35:** 781-788

Rost H, Malmstrom L, Aebersold R (2012) A computational tool to detect and avoid redundancy in selected reaction monitoring. *Molecular & cellular proteomics : MCP* **11:** 540-549

Rost HL, Liu Y, D'Agostino G, Zanella M, Navarro P, Rosenberger G, Collins BC, Gillet L, Testa G, Malmstrom L, Aebersold R (2016) TRIC: an automated alignment strategy for reproducible protein quantification in targeted proteomics. *Nature methods* **13:** 777-783

Rost HL, Malmstrom L, Aebersold R (2015) Reproducible quantitative proteotype data matrices for systems biology. *Molecular biology of the cell* **26:** 3926-3931

Sajic T, Liu Y, Aebersold R (2015) Using data-independent, high-resolution mass spectrometry in protein biomarker research: perspectives and clinical applications. *Proteomics Clinical applications* **9:** 307-321

Schmidlin T, Garrigues L, Lane CS, Mulder TC, van Doorn S, Post H, de Graaf EL, Lemeer S, Heck AJ, Altelaar AF (2016) Assessment of SRM, MRM and DIA for the targeted analysis of phosphorylation dynamics in non-small cell lung cancer. *Proteomics*

Schubert OT, Gillet LC, Collins BC, Navarro P, Rosenberger G, Wolski WE, Lam H, Amodei D, Mallick P, MacLean B, Aebersold R (2015a) Building high-quality assay libraries for targeted analysis of SWATH MS data. *Nature protocols* **10:** 426-441

Schubert OT, Ludwig C, Kogadeeva M, Zimmermann M, Rosenberger G, Gengenbacher M, Gillet LC, Collins BC, Rost HL, Kaufmann SH, Sauer U, Aebersold R (2015b) Absolute Proteome Composition and Dynamics during Dormancy and Resuscitation of Mycobacterium tuberculosis. *Cell host & microbe* **18:** 96-108

Selevsek N, Chang CY, Gillet LC, Navarro P, Bernhardt OM, Reiter L, Cheng LY, Vitek O, Aebersold R (2015) Reproducible and consistent quantification of the Saccharomyces cerevisiae proteome by SWATH-mass spectrometry. *Molecular & cellular proteomics : MCP* **14:** 739-749

Silva JC, Denny R, Dorschel CA, Gorenstein M, Kass IJ, Li GZ, McKenna T, Nold MJ, Richardson K, Young P, Geromanos S (2005) Quantitative proteomic analysis by accurate mass retention time pairs. *Analytical chemistry* **77:** 2187-2200

Silva JC, Gorenstein MV, Li GZ, Vissers JP, Geromanos SJ (2006) Absolute quantification of proteins by LCMSE: a virtue of parallel MS acquisition. *Molecular & cellular proteomics : MCP* **5:** 144-156

Teleman J, Karlsson C, Waldemarson S, Hansson K, James P, Malmstrom J, Levander F (2012) Automated selected reaction monitoring software for accurate label-free protein quantification. *Journal of proteome research* **11:** 3766-3773

Ting YS, Egertson JD, Bollinger JG, Searle BC, Payne SH, Noble WS, MacCoss MJ (2017) PECAN: library-free peptide detection for data-independent acquisition tandem mass spectrometry data. *Nature methods* **14:** 903-908

Ting YS, Egertson JD, Payne SH, Kim S, MacLean B, Kall L, Aebersold R, Smith RD, Noble WS, MacCoss MJ (2015) Peptide-Centric Proteome Analysis: An Alternative Strategy for the Analysis of Tandem Mass Spectrometry Data. *Molecular & cellular proteomics : MCP* **14:** 2301-2307

Tsou CC, Avtonomov D, Larsen B, Tucholska M, Choi H, Gingras AC, Nesvizhskii AI (2015) DIA-Umpire: comprehensive computational framework for data-independent acquisition proteomics. *Nature methods* **12:** 258-264, 257 p following 264

Venable JD, Dong MQ, Wohlschlegel J, Dillin A, Yates JR (2004) Automated approach for quantitative analysis of complex peptide mixtures from tandem mass spectra. *Nature methods* **1:** 39-45

Wang J, Bourne PE, Bandeira N (2014) MixGF: spectral probabilities for mixture spectra from more than one peptide. *Molecular & cellular proteomics : MCP* **13:** 3688-3697

Wang J, Tucholska M, Knight JD, Lambert JP, Tate S, Larsen B, Gingras AC, Bandeira N (2015) MSPLIT-DIA: sensitive peptide identification for data-independent acquisition. *Nature methods* **12:** 1106-1108

Weisbrod CR, Eng JK, Hoopmann MR, Baker T, Bruce JE (2012) Accurate peptide fragment mass analysis: multiplexed peptide identification and quantification. *Journal of proteome research* **11:** 1621-1632

Williams EG, Wu Y, Jha P, Dubuis S, Blattmann P, Argmann CA, Houten SM, Amariuta T, Wolski W, Zamboni N, Aebersold R, Auwerx J (2016) Systems proteomics of liver mitochondria function. *Science* **352**

Zhang Y, Bilbao A, Bruderer T, Luban J, Strambio-De-Castillia C, Lisacek F, Hopfgartner G, Varesio E (2015) The Use of Variable Q1 Isolation Windows Improves Selectivity in LC-SWATH-MS Acquisition. *Journal of proteome research* **14:** 4359-4371
